# Tumor microenvironment complexity and therapeutic implications at a glance

**DOI:** 10.1186/s12964-020-0530-4

**Published:** 2020-04-07

**Authors:** Roghayyeh Baghban, Leila Roshangar, Rana Jahanban-Esfahlan, Khaled Seidi, Abbas Ebrahimi-Kalan, Mehdi Jaymand, Saeed Kolahian, Tahereh Javaheri, Peyman Zare

**Affiliations:** 1 Drug Applied Research Center, Tabriz University of Medical Sciences, Tabriz, Iran; 2 Department of Medical Biotechnology, School of Advanced Medical Sciences, Tabriz University of Medical Sciences, Tabriz, Iran; 3 Stem Cell Research Center, Tabriz University of Medical Sciences, Tabriz, Iran; 4 Biotechnology Research Center, Tabriz University of Medical Sciences, Tabriz, Iran; 5 Student Research Committees, Tabriz University of Medical Sciences, Tabriz, Iran; 6 Department of Neurosciences and Cognitive, School of Advanced Medical Sciences, Tabriz University of Medical Sciences, Tabriz, Iran; 7 Nano Drug Delivery Research Center, Health Technology Institute, Kermanshah University of Medical Sciences, Kermanshah, Iran; 8 Department of Experimental and Clinical Pharmacology and Pharmacogenomics, University Hospital Tuebingen, Tuebingen, Germany; 9 Health Informatics Lab, Metropolitan College, Boston University, Boston, USA; 10 Dioscuri Center of Chromatin Biology and Epigenomics, Nencki Institute of Experimental Biology, Polish Academy of Sciences, Warsaw, Poland; 11 Faculty of Medicine, Cardinal Stefan Wyszyński University in Warsaw, 01-938 Warsaw, Poland

**Keywords:** Cancer cell interactions, Tumor microenvironment, Extracellular matrix, Cancer therapy, Stroma cell, Circulating tumor cells, Cell-free DNA, Apoptotic bodies, Exosome, Horizontal transfer, Cancer models

## Abstract

The dynamic interactions of cancer cells with their microenvironment consisting of stromal cells (cellular part) and extracellular matrix (ECM) components (non-cellular) is essential to stimulate the heterogeneity of cancer cell, clonal evolution and to increase the multidrug resistance ending in cancer cell progression and metastasis. The reciprocal cell-cell/ECM interaction and tumor cell hijacking of non-malignant cells force stromal cells to lose their function and acquire new phenotypes that promote development and invasion of tumor cells. Understanding the underlying cellular and molecular mechanisms governing these interactions can be used as a novel strategy to indirectly disrupt cancer cell interplay and contribute to the development of efficient and safe therapeutic strategies to fight cancer. Furthermore, the tumor-derived circulating materials can also be used as cancer diagnostic tools to precisely predict and monitor the outcome of therapy. This review evaluates such potentials in various advanced cancer models, with a focus on 3D systems as well as lab-on-chip devices.

Video abstract

Video abstract

## Background

The process of tumor formation and progression is influenced by two factors, namely genetic/epigenetic changes in the tumor cells and the rearrangement of the components of the tumor microenvironment (TME) through mutual and dynamic crosstalk [1]. TME consists of tumor cells, tumor stromal cells including stromal fibroblasts, endothelial cells and immune cells like microglia, macrophages and lymphocytes and the non-cellular components of extracellular matrix such as collagen, fibronectin, hyaluronan, laminin, among others [2, 3]. As the heart of TME, tumor cells control the function of cellular and non-cellular components through complex signaling networks to use the non-malignant cells to work for their own benefit. The consequence of such crosstalks is reflected in tumor formation and maintenance as well as deficient response to therapy and multi-drug resistance (MDR). The non-malignant cells in the TME are known to promote tumorigenesis in all phases of cancer development and metastasis [4, 5].

The source of intercellular communication is a complex network of cytokines, chemokines, growth factors, inflammatory mediators and matrix remodeling enzymes, but other fascinating mechanisms of interaction are now emerging. These include circulating tumor cells (CTCs), exosomes, cell-free DNA (cfDNA) and apoptotic bodies as novel horizontal gene transfer (HGT) mediators derived from tumor cells and delivering information to distant target cells including tumor cells and/or normal cells [6, 7].

Recent advances in tumor biology have shown that a comprehensive analysis of the multiple exchanges between tumor cells and their neighboring microenvironment is essential to understand the different underlying mechanisms of tumor growth and metastasis [8]. The loss of tissue integrity, carcinogenesis and further progress occurs as a consequence of reciprocal interactions between tumor cells with non-cellular (ECM) and cellular components of the TME [9, 10]. Therefore, on the other side of the argument, interactions in reactive non-neoplastic cells, genetically-altered tumor cells, and ECM control the majority of the stages of tumorigenesis effectively including clonal evolution, cancer heterogeneity, epithelial-mesenchymal-transition (EMT), migration, invasion, development of metastasis, neovascularization, apoptosis and chemotherapeutic drug resistance [11–14].

Due to the compelling role of TME in malignancy, many efforts are focused on this area [15, 16]. That is, a better understanding of the ways in which TME affects cancer progression is expected to make new targets available for the cancer cell isolation and cancer treatment. This can be achieved by interfering with the complex crosstalks established between cancer cells, host cells, and their surrounding ECM [10].

The recapitulating of TME is an important challenge in the development of experimental cancer models. In order to develop a reliable tool for personalized cancer therapy and drug development, it is essential to preserve the key characteristics of the original tumor. Recent advances on three dimensional (3D) platforms through the use of lab-on-chip and microfluidic devices [17] have provided an enormous opportunity to better stimulate the function and biology of TME and to bridge the translational gap between preclinical and clinical settings [18].

In this review, we look into the molecular interactions between cancer cells and their microenvironment and evaluate the effect of such interactions on the fate of cancer cells. The effect of tumor-derived circulating materials as novel cancer theranostics are also highlighted. To this end, we review the feasibility of implementing an innovative strategy pattern based on the interruption of these crosstalks to build an effective anti-cancer approach.

The cornerstone of the current review compared to the previous ones is its comprehensiveness. Previous reviews in this area are focused, for example, on recapitulating the gradual process of cancer metastasis by discussing advanced biomaterials and microtechnologies [19]. Also, they may highlight the mechanics of tumor metastasis [20]. And most of them only discussed a limited number of players/strategies such as anti-angiogenic therapies or targeting ECM yet fail to discuss the newly formed gadgets of cell-cell interactions such as cfDNA, apoptotic bodies, CTCs as well as exosomes [21, 22]. This review also evaluates the potential of disrupting tumor cells interactions in various cancer settings, in particular the newly emerging cancer models including 3D models and microfluidic platforms that allow to study different aspects of cancer cell behavior and biology, similar to the physiological environment in which they naturally occur.

## Mechanism of interaction

**Fig. 1:**
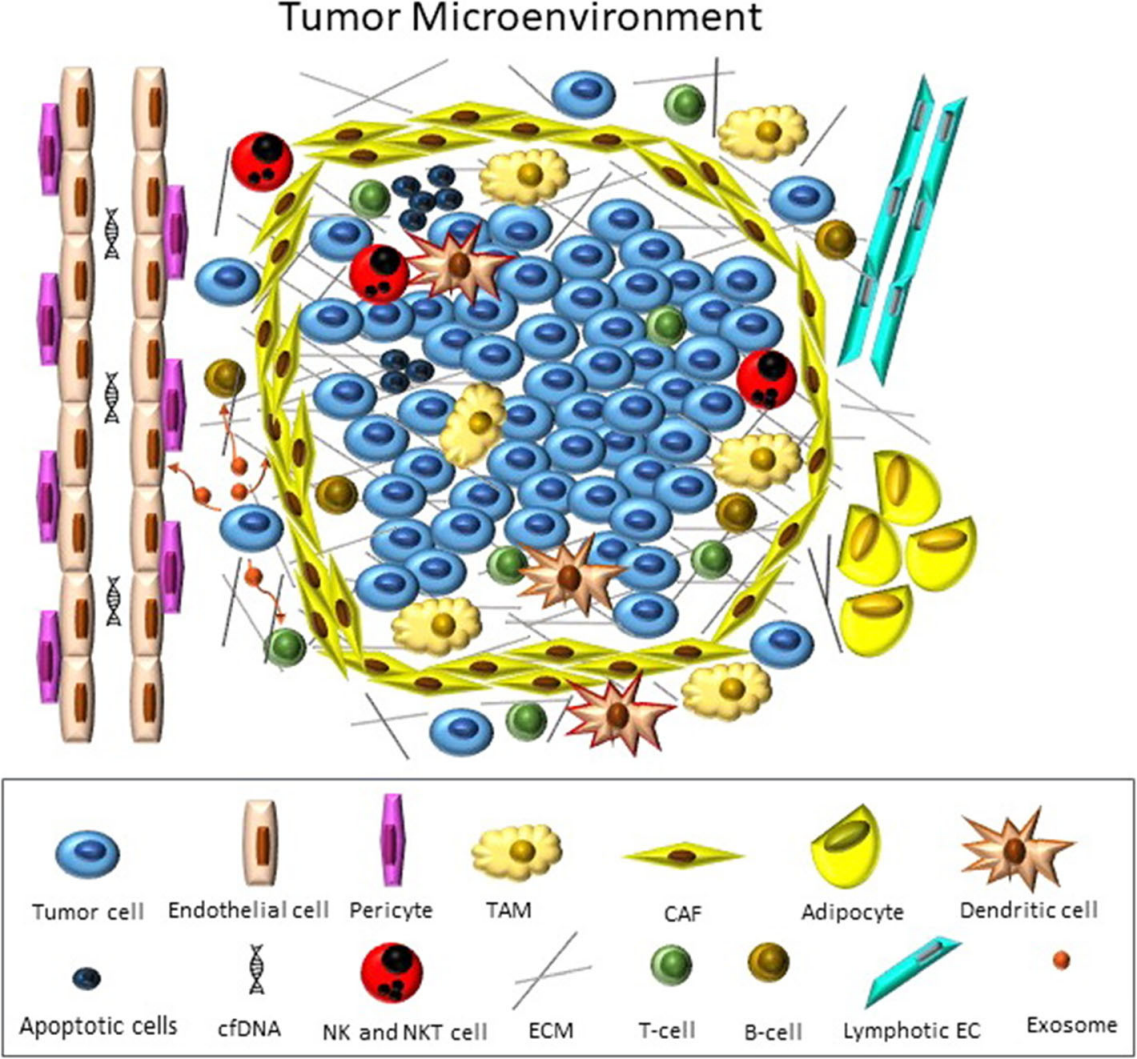
Tumor microenvironment at a glance. Tumor cells hijack different cellular and non-cellular non-malignant components of TME to promote their own growth and survival under hostile conditions. Meanwhile, the mediators for such contacts can be soluble factors (chemokines/cytokines/growth factors, etc.), or those that enable horizontal genetic/biomaterial transfer including cfDNA, apoptotic bodies, CTCs, and exosomes

Tumors develop in complex and dynamic microenvironments that influencetheir growth, invasion, and metastasis. In this space, tumor cells and their adjacent microenvironments are in frequent communication. The interaction of cancer cells with their microenvironment is dynamic and bidirectional and includes (i) cell-cell contacts, or cell-free contacts (involving ECM) and (ii) the mediators that enable these contacts. Mediators are secreted soluble molecules/factors/vesicles that are responsible for the horizontal transfer of genetic information between cellular/non-cellular communicating cells (Fig. 1).

## Understanding tumor cell interactions for effective cancer theranostics

Understanding the interaction between cancer cells can be used to develop therapeutic strategies to predict and neutralize tactics deployed by cancer cells to survive and resist anti-cancer modalities. Therefore, several strategies have been employed to combat these tumors by disrupting their interaction with stromal cells by anti-angiogenic therapy, immune modulation/reprogramming, CAF depletion, ECM targeting (e.g. collagen, hyaluronic acid depletion) and exosome/CTCs targeting.

**Fig. 2:**
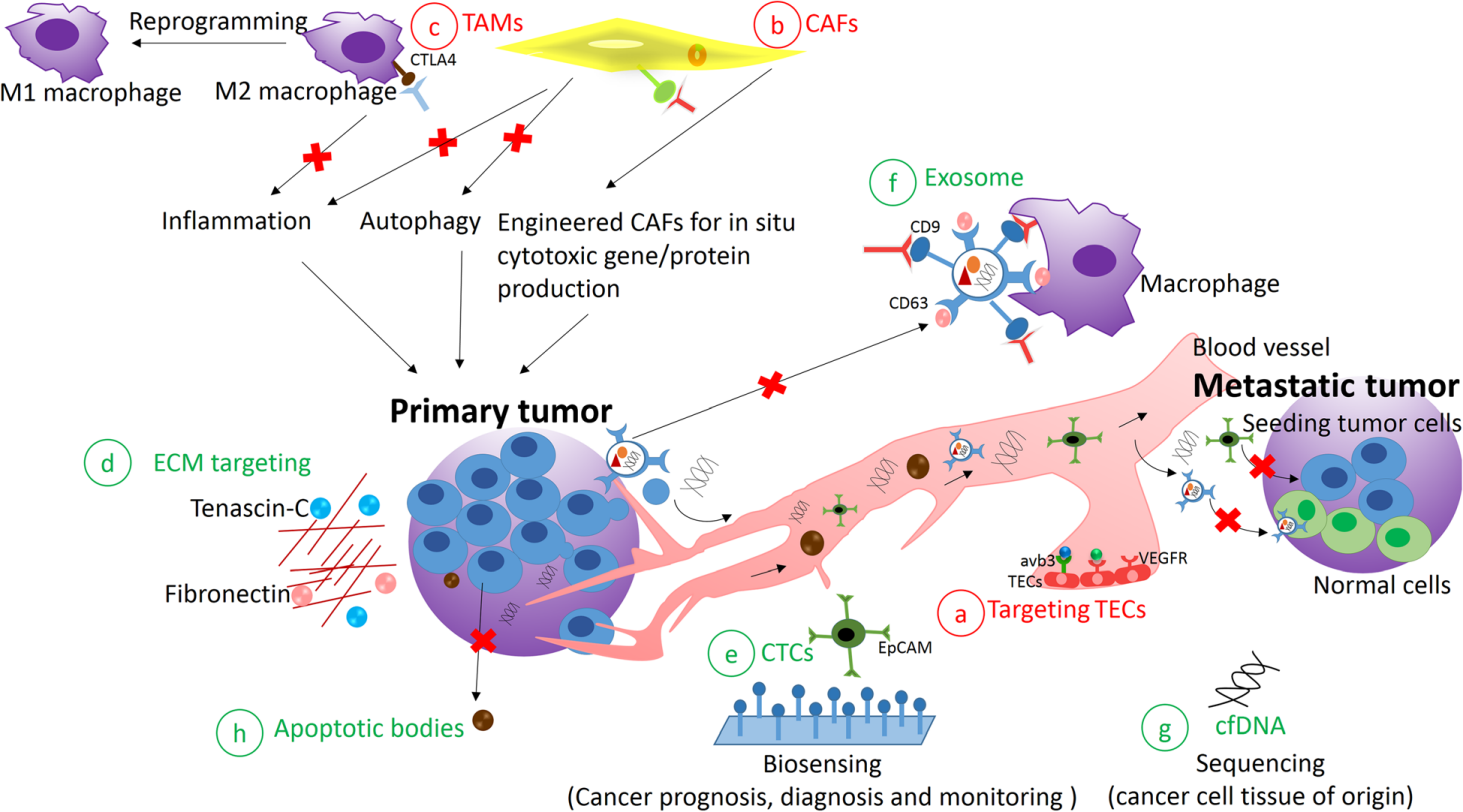
Exploiting different cellular and non-cellular components of TME for effective cancer targeting

In the meantime, the detection and monitoring of other tumor-interacting components, such as CTCs, cfDNA and apoptotic bodies in the bloodstream can be beneficial to extend the knowledge of the condition of the malignant disease and improve cancer detection and diagnosis, as well as enable timely and appropriate treatment (Fig. 2). To this end, the detection of low concentrations of these valuable biomarkers by non-invasive liquid biopsy in microfluidic devices and nano-biosensors are being actively perused and evolved. In the next section, we provide an overview of current therapeutic/diagnostic approaches based on the disruption/exploitation of tumor cell interactions by targeting either the contacts or the mediators in various advanced pre-clinical cancer models including 3D systems and lab-on-chip platforms.

### Pericytes

Pericytes are vital multifunctional cells in TME that envelop the surface of endothelial cells using cytoplasmic processes that extend along the abluminal surface of the endothelium [23]. Along with endothelial cells, pericytes are involved in the basement membrane remodeling during angiogenesis [24] and tumorigenesis [25]. In addition, pericytes have several functions in the immune system including the attraction of inborn leukocytes to exit blood vessels, regulating lymphocyte activation and eliciting direct phagocytic activity [26].

Due to their role in tumor angiogenesis, strategies targeting pericytes have been proposed as antiangiogenic therapies for cancer [27]. However, clinical trials have not yielded consistent conclusions [28, 29]. While several studies have shown that greater pericytes coverage corresponds to a better diagnosis [30], others indicated that therapeutic targets involving pericytes may exacerbate the process of tumor metastasis [31–34]. In this line, Semb et al. showed that in platelet-derived growth factor (PDGF) Bret/ret. mouse model, pericyte deficiency causes the spreading of metastatic insulinoma-derived cells [31]. Likewise, Kalluri et al. reported that genetic knockdown of tumoral pericytes in breast cancer upsurges pulmonary tumorigenesis in NG2-TK mouse model [34]. In contrast to these reports, it is shown that the production of pericyte by cancer cells promotes the growth of glioma tumor. In this regard, analysis of human specimens has revealed that glioblastoma stem cells (GSCs) are responsible for producing the majority of vascular pericytes to reshape the perivascular niche to support vascular function and tumor growth. GSCs migrate along the SDF-1/CXCR4 axis toward the ECs, where they are transformed into pericytes mainly by the action of the transforming growth factor β (TGFβ) [35].

Current strategies may lack sufficient specificity due to targeting of whole population of pericytes. A better understanding of the molecular mechanisms of tumor progression involving pericytes may reveal specific targets within pericyte subpopulations and thus contributes to cancer treatments [36]. This can be achieved in part by using microfluidic systems that can recapitulate more complex biological interfaces that would otherwise be unattainable by two-dimensional (2D) systems to study the dynamic interaction of pericytes with different TME components under normal or pathological conditions. For example, a 3D self-organized microvascular model of the human blood-brain barrier (BBB) with endothelial cells, pericytes, and astrocytes resembles the characteristic of a physiologic BBB similar to those of the rat brain, with perfusable and selective microvascularization and lower permeability than the conventional in vitro model [37]. Similar 3D lab-on-chip vascular network systems involving pericytes are discussed in [38, 39].

### Tumor endothelial cells (TECs)

In most solid tumors, endothelial cells (ECs) build up the inner layer of the blood vessels as part of a growing tumor [40]. Compared to normal ECs, tumor-derived endothelial cells (TECs) have a disturbed morphology and phenotypes at the cellular and molecular levels, similar to the tumor itself [40–42]. Considering their origin, tumor-derived endothelial cells (TECs) can be produced directly by differentiation of cancer cells, where they allow ECs to migrate into the tumor or eventually leave the tumor. However, in this review, TECs are ECs that benefit tumors, regardless of their location (in- or outside the tumor site) and/or their cell of origin. TECs are not only involved in the angiogenesis process to support primary tumor growth, but also promote tumor progression, metastasis and drug resistance [41]. TECs contain stem cell-like populations and overexpress MDR1 and aldehyde dehydrogenase (ALDH) and become resistant to chemotherapeutics such as paclitaxel and 5-fluorouracil in vitro [43, 44].

In addition, disturbed ECs offer a survival advantage to solid tumors. Since, disorganized TECs are essential for the characteristics of a tumor characterized by a leaking vascular system, high interstitial fluid pressure, reduced blood flow, tumor hypoxia and acidosis [45]. These properties promote the tumor cell heterogeneity, cancer resistance and impair efficient drug delivery [2, 8, 46]. Tumor hypoxia induces angiogenesis by activating the expression of vascular endothelial growth factor (VEGF) [3]. Meanwhile, TECs use various chemokine receptors (CXCR) including atypical chemokine receptor 1 (ACKR1), ACKR3, CXCR7, CXCR4, and chemokine (C-C motif) receptor 2 (CCR2) as markers of TECs to support tumor cells progression in numerous cancer types, recently reviewed in [40]).

As outstanding components of TME, TECs not only support the tumor with nutrients but also influence the immune cell infiltration and tumor’s stromal cell arrangement [47]. As proof of concept, it is documented that glioma-initiating cells located in the perivascular microenvironment are responsible for maintaining self-renewal capacity and glioma progression. Using a genetically engineered mouse model of PDGF-induced gliomas, it has been shown that this interaction was mediated through perivascular nitric oxide, which activates notch signaling to promotes stem-like character [48]. Besides notch activity, PDGF- nitric oxide synthase (NOS)-inhibitor of differentiation 4 (ID4)-miR129 axis are additional mediators involved in glioma progression [49].

Given the numerous supporting functions for TCs, TECs represents an indispensable target in cancer therapy. To this end, most primary strategies are directed at inhibiting tumor angiogenesis by blocking either growth factors or factors involved in endothelial cell migration, survival, and proliferation (Fig. 2a) [50] (see for review [3, 51]). Taken as an example of effective anti-angiogenic therapeutic approach, gold nanoparticles (NPs) are used to disrupt the signal transduction that wire TECs to CAFs or tumor cells. Mechanistically, gold NPs results in ∼95% VEGF165 removal from VEGF single-protein solution and deplete up to ∼45% of VEGF165 from conditioned media (CM) of ovarian cancer cells, as validated by decreased VEGF-receptor 2 (VEGFR2) activation compared to control CM. Thus, gold NPs block VEGF-VEGFR2 signaling from TME cells to endothelial cells and inhibit angiogenesis as reflected in reduced migration and tube formation of ECs when co-cultured with TCs in vitro [52].

Alternative approaches focus on the TECs, rather than on the TEC-derived growth factors. For example, one can target energy metabolism pathways in TECs, knowing that glycolysis-dependent for the synthesis of both biomass and adenosine triphosphate (ATP). Likewise, β-oxidation of fatty acids is indispensable for de novo nucleotide production during EC proliferation. In fact, inhibitors of these pathways can target and block proliferation and pathological angiogenesis in vivo [53]. Moreover, TEC can be used for cancer vaccine development. In this sense, Nomura et al. developed a dendritic cell (DC)-based immunotherapy, capable of targeting TECs. Prophylactic vaccination with DCs pulsed with lysates of TECs (positive for angiotensin-converting enzyme (ACE) activity) isolated from the lung with metastases was shown to significantly suppress lung metastasis in the B16/BL6 mouse melanoma model. DC-based vaccines that target TECs in tumor cells are likely to have effective therapeutic outcomes on distant metastasis [54].

Recent progress in 3D platforms have provided more insight into the critical roles of TECs and their collaboration with different TME components. 3D spheroids have provided a more reliable physiological environment for studying the interaction of TECs with tumor stroma, where the shape and surface texture of the spheroids indicates spatial invasiveness of cells in ECM [55]. In addition, 3D microfluidic system has enabled a high resolution, real-time imaging, and precise quantification of endothelial barrier function, which is essential for investigating the interaction of tumor cells during the metastasis process. The results indicated that secretion of tumor necrosis factor-alpha (TNFα) by macrophages can impair endothelial barrier as validated by a higher number and faster dynamics of TC-EC interactions in highly invasive fibrosarcoma cells [56]. Equally, 3D systems enable studying the organ-specific preference of metastatic cancer cells and the underlying molecular pathways/interactions. For example, a 3D vascularized organotypic microfluidic system is used to analyze organ-specific human breast cancer cell extravasation into muscle- and bone-containing matrices through a microvascular network concentrically wrapped with mural cells. The results indicated inhibitory role of adenosine on extravasation as blocking A3 adenosine receptors increased extravasation rates of breast cancer cells into the myoblast, mimicking microenvironments compared with untreated cells [57].

### Cancer-associated fibroblast (CAFs)

Cancer-associated fibroblasts (CAFs) in the immediate vicinity of cancer cells play an important role in tumorigenesis in various physicochemical ways by reducing apoptosis and improving the proliferation, migration and viability of cancer cells [58, 59]. CAFs residing in TME are heterogeneous cells with different origins, different functions (pro or anti-tumor activities) and different surface markers such as alpha-smooth muscle actin (α-SMA), myosin light chain 9 (MYL9), myosin light chain kinase (MYLK), matrix metalloproteinase 2 (MMP2), decorin (DCN) and collagen type I alpha 2 (COL1A2) [60–63].

Similar to their role in normal wound healing process, in the context of cancer, CAFs interaction with tumor cells occurs at several interfaces. CAFs produce ECM proteins, which prompts immunosuppression of tumor cells by recruiting immunosuppressive cells [64, 65] such as monocytes and inducing immunosuppressive PD-1+ TAMs, as recently shown in breast cancer cells in vitro [66]. In addition, CAFs promote angiogenesis by producing fibroblast growth factor 2 (FGF2), and vascular endothelial growth factor A (VEGFA) in different cancers [67] as well as galectin-1 expression in gastric cancer [68].

Moreover, CAFs positively influence the proliferation and metabolism of cancer cells through oxidative stress, which induces the autophagy pathway [69]. CAFs can also serve as nutrients for cancer cells, as oxidation of CAFs offers nutrients such as ketone and cytokines, which mediate mitochondrial biogenesis and autophagy, in nearby cancer cells [70, 71]. Furthermore, the CAF-derived cytokines CCL5 (chemokine ligand 5), IL6, and CXCL10 (C-X-C motif chemokine 10 regulate the metabolism of cancer cells by increasing phosphorylation of phosphoglucomutase 1 and glycogen mobilization, nicotinamide adenine dinucleotide phosphate (NADPH) synthesis and the tricarboxylic acid (TCA) cycle, which facilitates the proliferation and metastasis of ovarian cancer cells in vivo [72].

In addition to the findings from the regular cancer models, the 3D cancer models showed an obvious case of selective control of CAF function by TCs. A recent study on organoid and mouse models of pancreatic ductal adenocarcinoma demonstrated the opposing roles of tumor-secreted ligands including transforming growth factorβ (TGFβ) and interleukin 1 (IL1) to produce two distinct CAF subtypes characterized by either myofibroblastic or inflammatory phenotypes. In this report, activation of IL1/leukemia inhibitory factor (LIF)/janus kinase/signal transducers and activators of transcription (JAK/STAT) signaling generate inflammatory CAFs while TGFβ signaling antagonizes this process by downregulating interleukin 1 receptor, type I (IL1R1) expression and promoting differentiation into myofibroblasts [73]. Furthermore, tumor-stroma interactions are studied by 3D systems, in which CAFs derived from squamous carcinoma cells of the hypopharynx (FaDu) and head and neck cancer patients were incorporated into the tissue roll for the analysis of cellular environment and response (TRACER) platform. Results demonstrated that co-culture of CAFs with FaDu cells increased proliferation rate and invasive cell migration at 24 h and 48 h of culture with negligible effects on radiation resistance [74]. Moreover, in vitro organotypic microfluidic chip can be used to mechanistically investigate the TCs-CAFs interactions by co-culturing of breast cancer and patient-derived fibroblast cells in the 3D tumor and stroma regions, respectively. In this 3D model, CAFs were shown to enhance invasion and migration speed by inducing expression of a new candidate gene, glycoprotein nonmetastatic B (GPNMB) in breast cancer cells [75].

In view of the critical role of CAFs in the design of TME, the therapeutic options for deactivating CAF-mediated interaction are largely focused on using/reprogramming or eliminating of CAFs [76–78]. An example for the exploitation of CAFs is demonstrated in a study in which the off-target distribution of anticancer nanoparticles (NPs) to fibroblasts, that creates an obstacle to the effective management of desmoplastic tumors [79–82], was exploited to selectively deliver therapeutics cargos to cancer cells. In this preparation, NP damage is used for selective delivery of plasmid coding cytotoxic proteins (the secretable TNF-related apoptosis inducing ligand (sTRAIL) DNA complexes) loaded into liposome-coated protamine. A further experiment in xenograft model of human desmoplastic bladder carcinoma showed that this strategy led to 70% of CAFs as sTRAIL-producing cells. This was sufficient to remodel activated CAF into resting cells, meanwhile eliciting apoptotic effects on the nest of adjacent tumor cells. Thus the use of NP to modify CAFs can be an effective strategy to treat desmoplastic cancers (Fig. 2b) [83].

In the sense of blocking CAFs, in a recent study, Takai et al. reported Pirfenidone (PFD) has inhibitory effects on the viability of the cells and production of collagen in 2D culture media. It also suppressed the growth of tumor cells, mediated by CAFs, leading to apoptosis in 3D culture assay of 4 T1 tumor cells along with CAFs. PFD also suppressed metastasis in the lung and progression of the tumor in combination with doxorubicin in in vivo models [84]. Another strategy to inhibit CAFs is the use of specific antibodies, such as CAFs’ polyclonal rabbit anti-CAFs antibodies (poly Abs) achieved by immunizing rabbits with the bFGF-activated fibroblasts. Such polyclonal antibodies have been shown to delay tumor growth in mice bearing murine CT26 colon carcinoma [85].

Blocking autophagy in CAFs is another strategy to inhibit cancer cell proliferation. Drugs like metformin and gemcitabine are reported to induce autophagy. The combination of chemotherapeutic like α-cyano-4-hydroxycinnamate (CHC) alone and in combination with metformin is reported to hinder autophagic flux in CAFs and hampers tumor cell proliferation, irrespective of chemotherapeutic agents in in vitro and in syngeneic pancreatic cancer model [86].

### Tumor-associated macrophage

TAM is another key element of the TME which significantly affects cancer cell behavior [87]. Similar to CAFs, TAMs are heterogeneous and available in different types depending on their origin and function [88]. Based on their origin and half-life, they are (i) long-living embryonically (yolk sac)-derived tissue-resident macrophages and (ii) short-lived circulating monocyte-derived macrophages derived from bone marrow and recruited to tumor tissue by growth factors and chemokines, such as, CCL2, CCL5, and macrophage colony-stimulating factor (M-CSF) [89–91].

Considering their function, TME define remodeling of both infiltrating and resident macrophage into TAM. It has been recognized that TAMs may have both promoting (M1 type) and impairing roles (M2 type) when interfering with cancer treatments [92]. By producing migration-stimulating factors, TAMs give tumor cells the ability of motility and metastasis [93]. In this line, a study in human colorectal cancer (CRC) specimens and in vitro co-culture, revealed that TAMs induce EMT program to enhance CRC invasion, migration, and CTC-mediated metastasis by producing IL6 to activate JAK2/STAT3 axis and inhibit the suppressive role of miR-506-3p on FoxQ1 expression. This in turn leads to CCL2 production to promote macrophage infiltration. Blockade of IL6 or CCL2 demolishes this loop as evidenced by reduced mesenchymal CTC-mediated metastasis and macrophage migration, respectively [94]. Surprisingly, when interacting with apoptotic cancer cells in conditioned medium, TAMs inhibits TGFβ1-induced EMT and thus tumor invasion, which is considered the antitumor role of TAMs [95]. This newly discovered unusual action of TAM is discussed in the section of apoptotic cells.

Clinical studies and experimental animal models suggest that TAM commonly plays a pro-tumoral role in various ways [89, 96, 97]. For one, TAM strengthens angiogenesis by production of VEGF-A, TNFα, urokinase plasminogen activator (uPA), FGF, adrenomedullin (ADM), and semaphorin 4D (Sema4D) thymidine phosphorylase (TP), lymphangigensis (secretion of VEGF-A, VEGF-C, VEGF-D, CXCL8, MMP2, MMP9) fueling cancer invasion and metastasis [96, 98, 99]. Moreover, TAM-derived chemokines/cytokines (e.g. TGF-β, IL-6, IL-10, and TNF-α) is shown to enhance stemness of cancer cells by promoting EMT [100].

Accordingly, TAM-based therapeutic strategies are being developed that aim at TAM targeting, TAM re-education, and TAM depletion [88, 96, 101, 102].

It has been shown that TAM targeting in combination with immune control point inhibition achieves the best effects by administering blocking antibodies against inhibitory control point ligands such as programmed death ligand 1 (PD-L1) or receptors such as PD-1 and the cytotoxic T lymphocyte associated protein (CTLA4). TAM targeting, in turn, can be achieved by barricading the colony stimulating factor 1 receptor (CSF1R), which is essential for the recruitment, differentiation and survival of TAM [103] (Fig. 2c) [104]. Inhibitors of CSF1R can reduce TAM or cause phenotypic alterations that might hinder the growth and progression of cancer cells [92, 105–107]. This formulation is found as a promising approach in treatment of a variety of cancers including breast, lung, colon and melanoma in preclinical settings [92]. Targeting functional TAM molecules also provides an effective therapeutic strategy. An interesting example is to block Fc receptors on TAM which avoid depletion of anti-PD1 antibodies and therefore enhances the efficiency of the checkpoint therapy [92, 108]. However, repression of Fc receptor may impose overall immunosuppressive effects, since these receptors are expressed in various immune cells such as myeloid cells and cytotoxic lymphocytes [109].

Using 3D models more reliable information about the interaction of TAM with TME components at different stages of cancer development can be obtained and new drug targets identified. In this regard, a 3D ECM model has provided new insights into the role of TAM in tumor metastasis. It could be shown that TAM influences the migration rate of cancer cells by TGFβ1-induced MT1-MMP and the cancer cell migration persistence by the nuclear factor Kappa - light chain enhancer of activated B cells (NF-κB)-dependent MMP1 expression. Thus, dual targeting of both pathways can be applied to effectively mitigate macrophage-induced metastasis [110].

TAM depletion by induction of selective activation of apoptosis pathways in TAM by agents such as alendronate-glucomannan conjugate [102] and TAM re-education to convert macrophage to M1 phenotype [101], as well as use of TAM as carrier for selective drug delivery to cancer cells are additional therapeutic tactics [10, 111, 112].

### ECM

ECM forms the scaffold of tissues and organs through the production of supramolecular aggregates, such as fibrils and sheet-like networks [113, 114]. It is a complex network composed of fibrous proteins (collagen, elastin), glycosaminoglycans (hyaluronic acid), proteoglycans (chondroitin sulfate, heparan sulfate), and glycoproteins (fibronectin 1 (FN1), laminins, tenascin C (TNC)) [115, 116]. ECM proteins can be produced by many stromal cell types and tumor cells, however, CAFs are the main source for synthesis, secretion, assembly, and modification of the ECM composition and organization [60, 117].

Besides its biochemical composition, such as intermolecular covalent cross-linkages, the ECM biophysical characteristics include its topography, stiffness/rigidity, molecular density, and tension [118]. Therefore, ECM is very versatile and is subject to remodeling, which is under the influence of tumor stroma, and cancer cells [119]. Dynamic crosstalk is mediated by growth factors, chemokines and metastatic CTCs tethered to and released from the ECM, as well as metabolic changes of the cells within the tumor bulk [120, 121]. ECM may act as a barrier for drug delivery through increased tissue stiffness and desmoplasia or a gate for breaching the basement membrane to promote metastasis [2, 122]. Furthermore, the ECM of distant organs can be remotely shaped into permissive/restrictive soils by soluble factors/CTCs/exosomes from primary tumors to facilitate the seeding of metastasizing cancer cells (See for review [9]).

Each component of ECM plays an important role in the cancer progression. Among them, the role of collagen stands out. Synthesis of collagen can be regulated by cancer cells, mutated genes, signaling pathways/receptors, and transcription factors [123]. Collagen in turn influences tumor cell behavior through integrins, tyrosine kinase receptors, discoidin domain receptors, and some signaling pathways [124]. Other partners in close contact with collagen involvement in cancer are microRNAs (miRNAs) [125] and exosomes [126]. Moreover, collagen interaction with other ECM molecules including fibronectin, laminin, hyaluronic acid, and MMPs influences cancer cell activity [127]. In addition, hypoxia, which is common in collagen-rich tumors, promotes cancer progression [124].

A deep understanding of the contribution of collagen to tumor progression can be achieved using 3D models. In this context, the role of desmoplasia and stromal fibroblasts on anti-cancer drug resistance is being investigated, wherein highly invasive breast cancer (MDA-MB-231) were embedded in microwells surrounded by CAFs encapsulated within collagen I hydrogel. Combined administration of tranilast (anti-fibrotic drugs) and doxorubicin significantly diminished tumor growth and invasion, as validated by reduced stiffness of the stromal matrix, disrupted fibronectin assembly and reduced collagen fiber density [128]. Also, bi-transgenic tumor model confirmed the role of stromal collagen condensation as indication for mammary tumor initiation and progression. Furthermore, studying epithelial-stromal interactions in normal mammary glands, mammary tumors, and tumor explants in 3D culture revealed the role of collagen reorganization at the tumor-stromal interface to facilitate local invasion [129].

Therapeutic options can be planned to treat each component of ECM. For example, LOX enzymes are widely used to block collagen crosslinking in various preclinical settings [130, 131]. Alternatively, ECM components can be used to ensure a precise tumor drug delivery [132]. Tenascin-C, a300 kDa large glycoprotein, is overexpressed within the ECM of numerous cancer cells such as breast, colon, lung, and ovarian. Its concentration in healthy ECM is almost low-to-none [133]. It also derive proliferation, angiogenesis and metastasis stages of tumor progression [134]. Dal Corso et al. introduced a study in which a non-internalizing antibody against tenascin-C was exploited to transport anthracycline (PNU159682), a chemotherapeutic agent, into the ECM of cancer cells (Fig. 2d). In the case of intravenous injection, the antibody-drug conjugate was found to bind to tenascin-C and the drug was discharged when the protease-sensitive linker between the antibody and the drug was cleaved. This in turn resulted in significant tumor growth inhibition of epidermoid carcinoma mouse xenografts [135]. Likewise, Chen et al. designed liposomes bound to tenascin-C peptide, as well as being loaded with navitoclax, a tiny molecule capable of causing apoptosis in CAFs. Such liposomes were capable of regulating the tumor ECM through efficiently removing CAFs, rendering the ECM accessible for doxorubicin-loaded nanoparticles that were administered later [136].

Chemotherapeutic agents can also be targeted to ECM of the tumor cells through the membrane-bound receptors such as tenascin-C [132]. In an interesting study, immune checkpoint inhibitors (antibodies) including anti-CTLA4 and anti-PD-L1 plus IL-2 were conjugated to the collagen-binding domain of the blood protein von willebrand factor (VWF) A3 domain to reduce side effects. This formulation allowed drug targets to bind to the tumor stroma to exert their effects locally and gained a promising efficacy and safety profile compared to the unconjugated molecules tested in several mouse models. Importantly, combination treatment with CPI and IL-2 resulted in complete elimination of tumors in a considerable number of animals (9 of 13) bearing orthotopic breast cancer [137].

Other ECM components with therapeutic value include fibronectin extra domain A and B (anti-EDB aptide) [138], laminin (IKVAV) [139], gelatin (anginex, small geletic-1 binding peptide) [140], aggrecan (a conjugate of quaternary ammonium) [141], and heparan sulfate (CGKRK peptide), among others [142].

### Circulating tumor cells

Cancer cells that are detached from the primary tumor site and entered the bloodstream are categorized as CTCs [143]. The implication of CTCs is well established in tumor cell dormancy, as a major cause of metastatic outgrowth, multi drug resistance (MDR) and cancer relapse [144]. These cells are precise representations of primary and metastatic tumors that convey information for the detection, diagnosis (monitoring) and the treatment of cancer [145]. Notably, even confined tumors without metastasis can produce CTCs [146]. Thus, they can serve as valuable prognostic, diagnostic, and biosensing tools to detect cancer cells that are clinically undetectable, and to make plans for timely and precise therapeutic interventions (Fig. 2e) [9].

Even more interesting is that, in contrast to the traditional view that tumor cell metastases occur unidirectional, the reverse process is also possible through CTC-mediated “self-seeding”. In this way, aggressive CTCs preferentially mediate self-seeding of breast, melanoma, and colon cancers in mice, including those with bone, brain, or lung -metastatic tropism. Mechanistically, tumors secrete IL-6 and IL-8 cytokines to attract CTC while CTC infiltration into mammary tumors is mediated by MMP1/collagenase-1 and the actin cytoskeleton component fascin-1. Consequently, tumor self-seeding leads to enhanced angiogenesis, tumor growth, stromal recruitment through seed-derived factors including the chemokine CXCL1, anaplasia, tumor size, and vascularity. Finally, there is the prognosis for local recurrence by seeding of disseminated cells after apparently complete tumor ablation [147, 148].

Contrasting results are reported on the efficiency of various treatments for reducing CTCs. Martin M. et al. assessed the variations in CTCs in 117 breast cancer patients and observed a considerable decline in CTC-positive rate after chemotherapy [149]. Likewise, Rack B. et al. piloted a larger prospective study with 2026 breast cancer patients and discovered that the detection rate of CTCs after chemotherapy (22.1%) increased slightly in comparison to the baseline condition (21.5%) [150]. Another study, involving 6712 breast cancer patients showed a decrease in the number of CTCs only in human epidermal growth factor receptor 2 (HER2+) or HER2- patients but not in the triple-negative ones and nor in patients who underwent surgery. Therapeutic regimes including metastatic treatment, adjuvant treatment, neoadjuvant treatment, or combination therapy were equally effective in reducing CTC positivity and the chance of disease progression [151]. In summary, despite uncertainty about the analytic validity, clinical validity, and clinical utility of CTCs, their status is a valuable indicator of the efficacy of cancer therapy, which may aid clinicians to make decisions for further personalized therapy.

Since an increase in the number of CTCs is associated with a poor prognosis, CTC-targeted therapies may provide a new approach to improve the prognosis of cancer therapy [152]. CTCs usually express simultaneously over one immune checkpoint [153], hence the blockage of multiple immune checkpoints can immediately confine more biomarkers among CTCs with higher immune recognition avidity. In this sense, Lian et al. described a unique set of checkpoints, CD274 and CD47, on CTCs which camouflaged these cells from being detected by immune cells and corresponding apoptosis. Their results indicated that simultaneous administration of anti-CD274 antibody (also known as PD-L1 or B7-H1) and anti-CD47 checkpoints antibody can respectively block the signal of “don’t find me” for immune evasion and “don’t eat me” for phagocytosis on CTCs. Thus, these combination shifts immune evasion to immune activation, which resulted in enhanced anti-tumor growth activity and reduced CTCs metastasis in 4 T1 tumor mouse model in vivo [153].

**Fig. 3:**
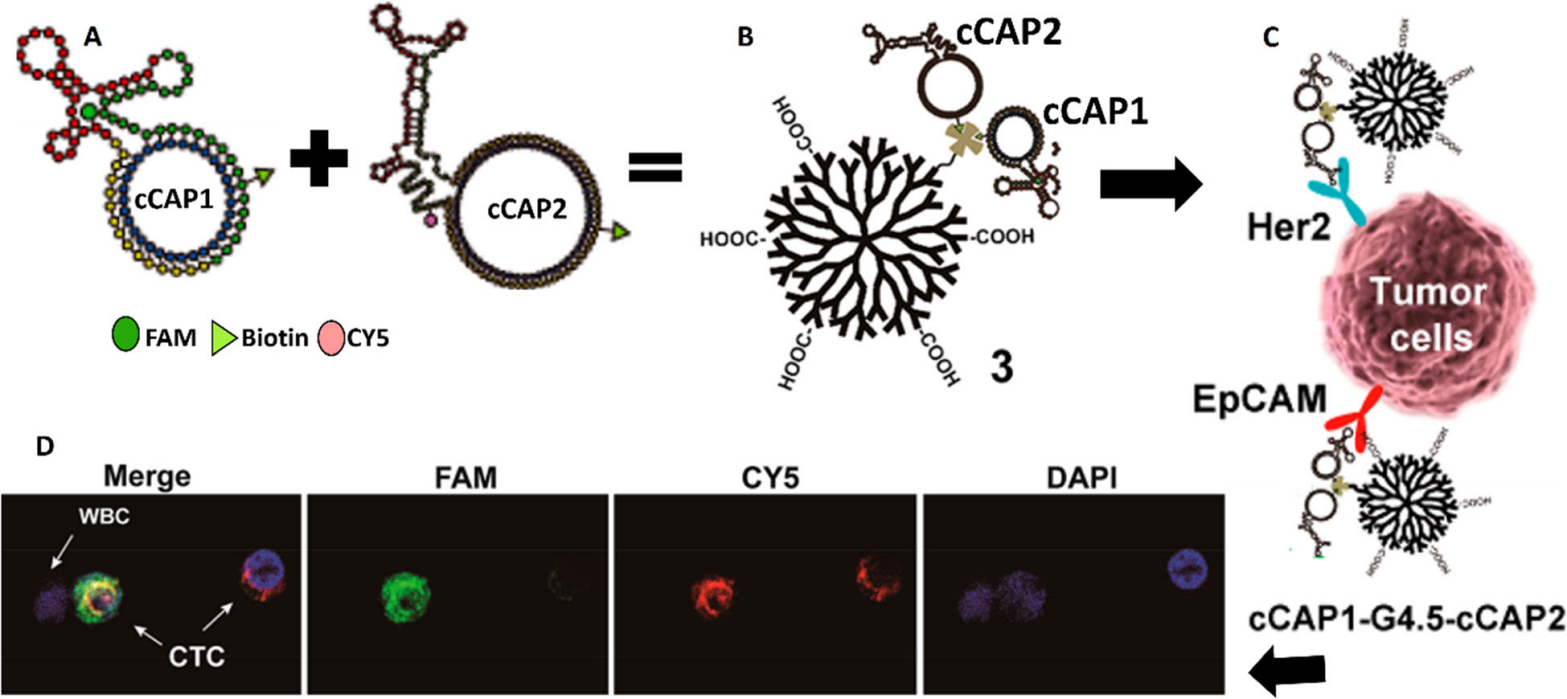
Design and working principle of aptamer-dendrimer (G4.5) nanomaterial for dual targeting of CTCs to reduce cancer metastatic burden. A, schematics of ring aptamers cCAP1 for targeting EpCAM and cCAP2 for targeting Her2 on CTCs. B, C, construction of aptamer ring conjugate (cCAP1-G4.5-cCAP2) for simultaneous binding and capturing of two CTC markers. D, Ex vivo analysis of CTCs in the blood of breast cancer patients captured by cCAP1-G4.5-cCAP2. Adapted with permission from [154]. Copyright (2017), American Chemical Society

Dong et al. reported on the design, synthesis, and description of the new dual double-stranded (ds) aptamer ring conjugate (cCAP1-G4.5-cCAP2) capable of simultaneously targeting EpCAM and Her2 epitopes on CTCs. This aptamer-conjugate can work properly when 10^8^ interfering cells or blood cells that do not express EpCAM or Her2 are present, as well as in complex biological samples of patients and mice with greatly enhanced bio-stability and high capturing precision. The aptamer-conjugate impeded metastasis and displayed enhanced bio-stability against endogenous nucleases in vivo. In this formulation, capture arms could distinctly bind two biomarkers at the same time (EpCAM and Her2). This, induced apoptosis as a result of the arrestment of cell cycle and the inhibition of tumor progression in captured CTCs (Fig. 3) [154].

**Fig. 4:**
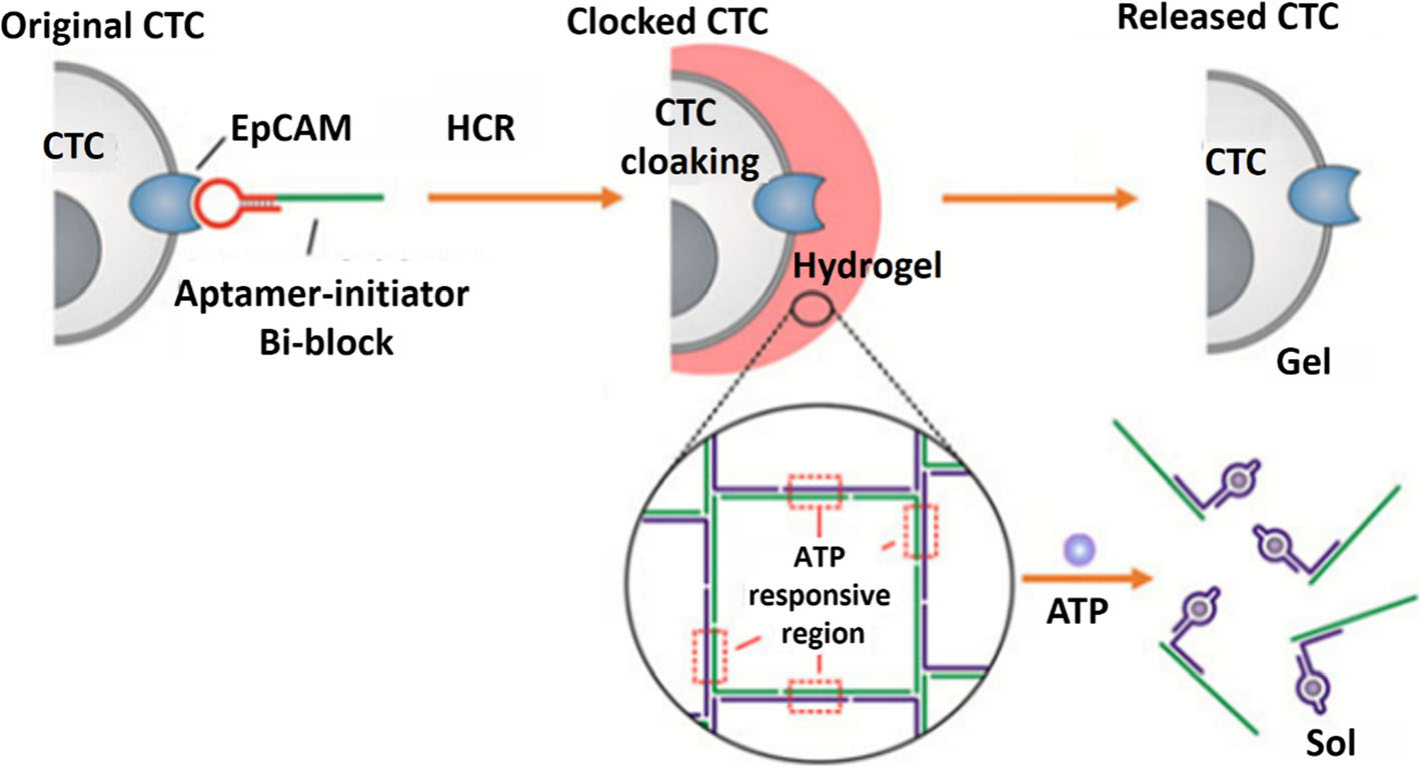
CTCs as valuable diagnostics for cancer management. DNA hydrogel-based liquid biopsy provides a highly sensitive platform for isolating CTCs expressing EpCAM and enables further live analysis with minimal damage. Adapted with permission from [156]. Copyright (2017) American Chemical Society

Given that CTCs can be used as diagnostic tools providing molecular information on the primary tumor state, the development of liquid biopsy platforms capable of capturing this rare population of cells from the blood is highly rewarding [155]. In this line, an interesting formulation based on DNA hydrogel is developed in which a DNA staple strand with aptamer-toehold biblocks binds to EpCAM receptor. This CTC-specific binding, initiate aptamer-triggered clamped hybridization chain reaction via toehold-initiated branch migration on CTC surface, realizing single/clusters of live CTC clocked in DNA hydrogel. The hydrogel is ATP-responsive which allows further stimuli-responsive shifting of gel to sol state, to decloak and release CTC for live cell analysis (Fig. 4) [156].

Upregulation of EphA2 occurs in several cancers such as melanoma [157], ovarian [158, 159], prostate [160, 161], lung [162, 163], and breast [164, 165] cancer. In a recent study, Wang et al. developed a peptide–drug conjugate (PDCs) using EphA2 agonists, YSA peptide or its enhanced version, 123B9. Their studies suggested that YSA– and 123B9– drug conjugates could selectively transport cytotoxic drugs to cancer cells in vivo [166, 167].

Furthermore, a dimeric 123B9 was able to perform receptor activation at concentrations in the nanomolar range. Additionally, dimeric 123B9 conjugation with paclitaxel is found to be very useful in combating CTCs and preventing the lung metastasis in breast cancer models [168].

### Exosomes

Multi-vesicular body (MVB)-derived extracellular vesicles (EVs) are constantly secreted into the extracellular space. These nanoparticles called exosomes are key to maintain homeostasis of their releasing (originating) cells [169–172]. They facilitate specific cell-cell interactions and stimulate several signaling pathways in their target cells, including cancer cells [173]. The production and release of exosomes from the tumor cells transmits a great deal of information with regards to the molecular and genetics properties, from the tumor cells to healthy ones or other abnormal cells residing nearby or at distant sites specifically designed to promote tumor invasion, metastasis and drug resistance [174]. That, bidirectional transport of exosomes containing different species of RNA (e.g. miRNA) and proteins between cancer stem cells and the fibroblast-rich microenvironment is shown to promote the growth of the tumor and the metastatic outbreak in breast carcinoma models [172]. Besides, exosomes are involved in acquired drug resistance, as evidenced by the observation that the transfer of the oncogene MET by exosomes modify surrounding icotinib-sensitive cells to promote icotinib-resistant lung cancer cells, that produce MET-containing exosomes and elicit the migration and invasion properties in vitro [175].

Ever since Stephen Paget’s 1889 hypothesis, the underlying mechanism for metastatic organotropism, the organ-specific homing of metastatic tumor cells on secondary sites was unknown. The discovery of exosomes and uncoupling their roles has opened a new paradigm in understanding preferred cancer cell interactions. Now, new discoveries point to the role of exosomes as media for predicting organ-specific metastasis. In this perspective, exosomes from mouse and human lung-, brain- and liver-tropic tumor cells fuse preferentially with resident cells namely lung fibroblasts and epithelial cells, brain endothelial cells and liver Kupffer cells at their predicted destination. Furthermore, uptake of tumor-derived exosomes prepares organ-specific cells to serve as pre-metastatic niche. Notably, the injection of exosomes from lung-tropic models can redirect the metastasis of bone-tropic tumor cells. Moreover, these exosomes display differential integrin expression, and blockade of integrins α_v_β_5_ and α_6_β_4_ dampen exosome uptake, as well as liver and lung metastasis, respectively. Finally, integrin-mediated uptake of exosome activates Src phosphorylation and pro-inflammatory S100 gene expression in resident cells [176].

Another interesting role of the exosome as mediators of cancer cell interaction with non-malignant cells is the observation that cancer cells can systemically reprogram energy metabolism by recipient premetastatic niche cells to promote metastasis. To increase nutrient availability, breast cancer cells secrete miR-122 -enriched vesicles to suppress glucose uptake by niche cells in vitro and in vivo. Mechanistically, miR-122 downregulate the glycolytic enzyme pyruvate kinase and restores glucose uptake by lungs and brain and lessen disease progression [177].

Form therapeutic point of view, exosomes are applied as diagnostic biomarkers, therapeutic targets, or as anticancer drug-delivery vehicles [178]. Communications mediated by exosomes in cancer can be disrupted by inhibition of the exosome production, secretion, cell-to-cell communication as well as removal of exosomal-specific loads [179, 180]. Also given their stability in systemic circulation and tumor-based specificity, exosomes are studied for their abilities to deliver anti-cancer agents [181].

A number of preclinical and clinical findings have reported that targeting pathways and molecules involved in the formation of exosome can inhibit tumor progression, such as heparanase/syndecan-1 axis [182, 183] or syndecan heparan sulfate proteoglycans [179, 184]. Sento et al. reported that heparin may suppress metastasis by lessening the uptake of exosomes, derived from cancer cells in oral squamous cell carcinoma [185]. In addition, Nishida-Aoki and colleagues applied therapeutic antibody targeting EVs including human-specific anti-CD9 or anti-CD63 antibodies which aimed at decreasing tumor-derived exosomes generation, possibly through clearance of EVs by macrophages, resulting in a decline in breast cancer distant metastasis in a mouse model (Fig. 2f) [186].

**Fig. 5:**
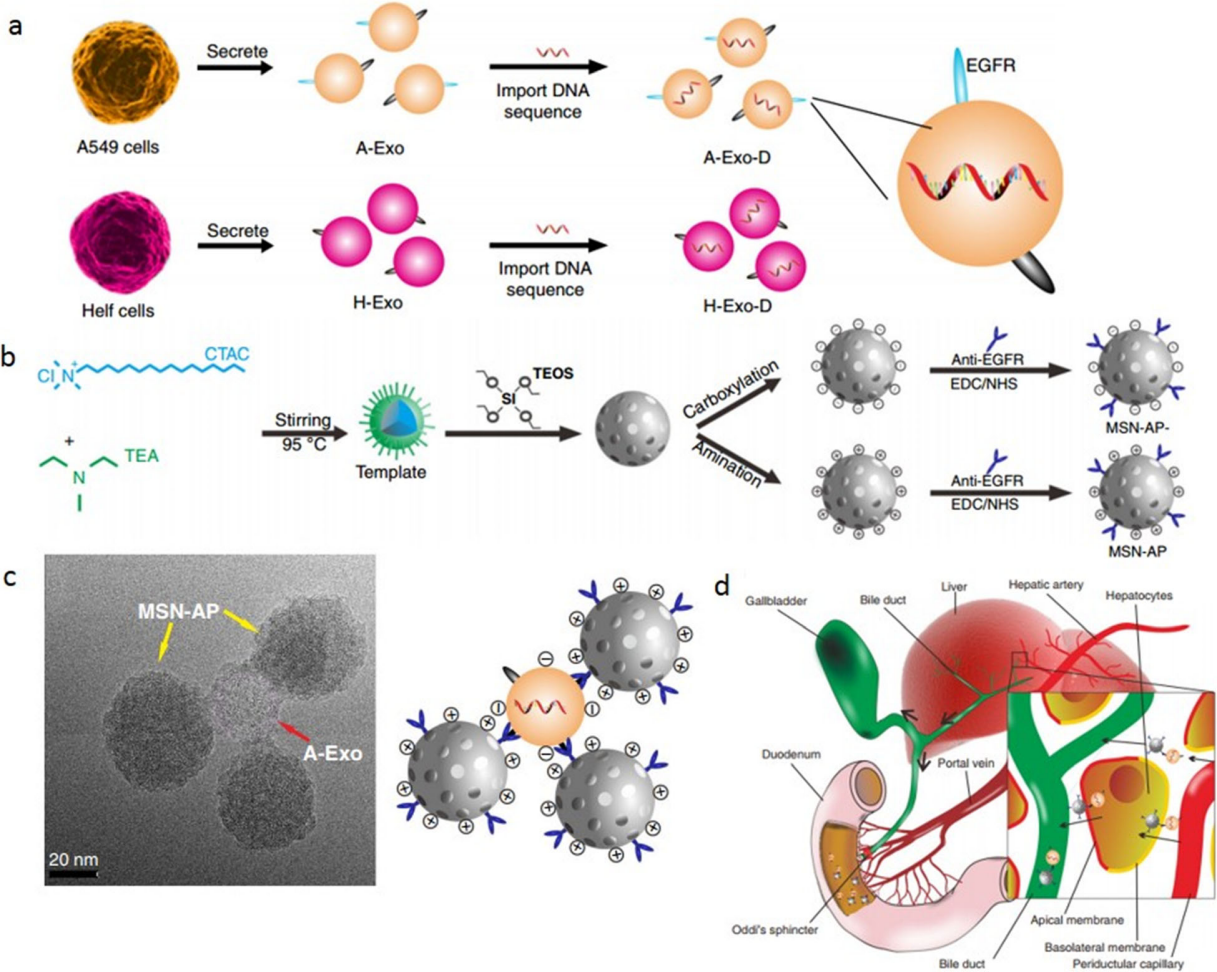
A schematic representation of the exosome preparation. a-b, Exosomes derived either from the human lung cancer cell line A549 (A-Exo) with high EGFR expression or from the human lung fibroblast cell line HELF (H-Exo) with low EGFR expression. The isolated exosomes were further transfected with a DNA sequence coding for CD9 and CD63 markers. Synthesis and functionalization of MSN with EGFR-targeting aptamers. c, A-Exo is recognized and captured by MSN-AP in cell media and rat blood. d, MSN-AP eliminates circulating exosomes in animals and patient blood. Adapted from Springer Nature: *Nature Communication*, Copyright (2019) [188]

In an interesting report, M-Trap is reported as an exosome-based device for capturing metastatic tumor cells. In this study, a synthetic pre-metastatic niche loaded with tumor exosomes in a 3D system was designed and then implanted in the peritoneum of the mouse. This formulation resulted in the deviation of ovarian tumor cells into the device, thereby capturing the cells and inhibiting further tumor metastasis [187]. Another interesting exosome-trapping system is reported that can specifically recognize, drag and dump blood-borne A549 lung cancer cell derived-oncogenic exosomes (A-Exo) into small intestine. In this design, EGFR-targeting aptamers coated on positively charged mesoporous silica nanoparticles (MSN-AP) can specifically identify and bind negatively charged A-Exo. Nanoparticle-A-Exo conjugates can cross hepatobiliary layers and Oddi’s sphincter into the small intestine, as validated by significant drop in circulatory A-exo, higher accumulation in the intestine and decreased lung metastasis in mice (Fig. 5) [188].

Furthermore, exosomes are the best candidates for gene therapy and the targeted drug delivery purposes, as they are natural, non-immunogenic, biodegradable, non-toxic, and more importantly capable of engineering for targeted therapy. Accordingly, exosome-producing cells can be fabricated to express and display transmembrane-anchored tumor-specific ligands on the surface of the exosome. In this regard, Limoni et al. fashioned exosomes conjugated with the chimeric protein against HER2+ cancer cells. To this, the transduction of HEK293T cells was performed by a lentiviral vector carrying-LAMP2b-DARPin G3 chimeric gene, where lysosomal associated membrane protein (LAMP) was served as anchoring chimerization with the ligand (DARPin for Her2 targeting) [189]. These exosomes delivered therapeutic siRNA into the targeted breast cancer cell lines resulting in 70% decrease in TPD52 gene expression in SKBR3 cells [190]. More recently, exosomes have also been targeted to deliver DOX to HER2+ cancer cells to evaluate the anticancer effects of DOX-loaded targeted exosomes in a murine tumor model. The results of this study indicate that targeted exosomes are favorably uptaken by HER2+ cells compared with HER2− cells and have the potential to be used as a competent drug delivery system [191].

### Circulating free DNA

Circulating/cell-free DNA (cfDNA), a cell-free nucleic acid, is produced from dead, necrotic and living eukaryotic cells [192]. It consists of very short (< 200 bp) double-stranded DNA fragments obtained at very low concentrations [193–195]. In cancerous conditions, cfDNA is derived not only from the cancer cells but also from TME and other non-cancer cells, e.g., endothelial and immune cells [196, 197]. Nevertheless, cancerous conditions that are marked with the increased cfDNA concentration and a significant amount of cfDNA are likely to be derived from the tumor, hence offering diagnostic evidence (Fig. 2g).

Notably, cfDNA fragments are able to enter neighboring/distal cells and are capable of altering the biology of recipient cells. In the context of cancer, they are involved in horizontal gene transfer and oncogenic transformation of normal cells as well as in the metastasis development [197]. Although there is no clear mechanistic evidence for their oncogenic potential, several proposals include (ii) overexpression of several pro-metastatic genes through the toll-like receptor 9 (TLR9)/ myeloid differentiation primary response 88 (MYD88) independent pathway [198]; (ii) transposable elements [199] and finally the cellular uptake of exosomes [200, 201]. Another consequence of cell-free contacts involving cfDNA is the increased chemo-, radioresistance of cancer cells, as radiotherapy produces oxidized DNA which triggers reactive oxygen species and induces DNA damage response pathways [202].

DNA released by leukemic cells in the form of nucleosome-like complexes can disrupt bone marrow (BM) structure and kill stromal cells by inducing genomic instability and induction of apoptosis. Mechanistically, entry of DNA into the nuclei of BM or other cells induce H2A.X phosphorylation at serine 139, similar to double-strand break-inducing agents, which induce killing of cells in a concentration-dependent manner in vitro and in vivo [203]. Furthermore, cell-free chromatin (cfCh) from dying cancer cells is able to integrate into the nuclei and genomes of non-malignant cells (NIH3T3 mouse fibroblast cells) in mice. The uptake of cfDNA induce the oncogenic transformation of bystander cells both locally and in distant organs, resulting in metastasis through a process referred to as “genometastasis” [204, 205]. Genometastasis is shown to occur by induction of two linked pathologic features of ontogenesis, DNA damage and inflammation as confirmed by activation of H2A histone family member X (H2AX) and inflammatory cytokines NFκB, IL-6, TNFα and interferon gamma (IFNγ) [206].

From another perspective, cfDNA can also indicate genetic and epigenetic characterization of tumors cells with minimally non-invasive means, simply from the blood plasma and serum of the patients [207, 208]. These methods can also eliminate the need for biopsy, additionally providing mutation-related information that allows easy monitoring of the tumor and provides therapeutic potential [209]. Furthermore, cfDNAs contain information on the mutations that affect treatment, facilitate individualized therapeutic examining, and non-invasive follow-up that may enable better cancer management [210]. However, the extraction and amplification of cfDNA can be demanding due to high DNA fragmentation and low concentration in the bloodstream [211].

cfDNA can be specifically used when tumor tissue is unavailable or insufficient for testing [212]. Liquid biopsy, intended to monitor cancer treatment responses has recently been considered to be a promising non-invasive cancer-related test that puts cell-free tumor DNA to use [213, 214]. Moreover, cfDNA is now recognized as a biomarker of absolute novelty in cancer diagnosis. A substantial number of strategies for breast cancer detection by cfDNA exist, including cfDNA concentration- as the initial quantitative detection method for breast cancer- cfDNA integrity, microsatellite alteration, gene mutations, and DNA methylation, among others [215]. What’s more, recently, a machine-learning model that uses the fragmentation pattern of cfDNA across the genome is shown to be capable of revealing tissue-of-origin of seven different cancers at detection sensitivity of 57% to more than 99% with 98% specificity. Also, it can detect 91% of patients with cancer when combined with mutation-based cell-free DNA analyses [216]. Therefore, with the advancement of biosensing technologies, application of cfDNA can be further extended to disease monitoring with promising prophylactic and diagnostic potential.

### Apoptotic bodies

Apoptotic bodies belong to the category of EVs that are released from the cells undergoing apoptosis and are known to have immunoregulatory properties in pathological conditions such as cancer [217]. There are markers to track phagocytosis of the cells and their uptake by phagocytes and/or cells nearby. Intracellular contents are packaged into membrane-bound apoptotic bodies, thus bypassing unwanted inflammatory responses such as the release of self-antigens into the surroundings. Nuclear fragments that are not engulfed by apoptotic bodies act as self-antigen and are the source of the autoimmune system-related diseases, e.g. systemic lupus erythematosus [218–220].

Apoptosis produces many apoptotic bodies containing a broad spectrum of cell components including DNAs, mRNAs, miRNAs, proteins, and lipids. Following the engulfment of apoptotic bodies by different cell types (macrophages, epithelial cells, fibroblasts, dendritic cells, and endothelial cells) subsequent internalization, devouring, and destruction of corpses occur in the lysosomes [221]. Since apoptotic cell engulfment could cause the generation of molecular memory by macrophages, apoptotic bodies are thought to accelerate intercellular communication via transferring cellular factors [222]. The entry of apoptotic bodies is thought to play important role in genetic alteration and diversity of the tumor cells. Further, the horizontal transfer of DNA might cause changes in the genetic information leading to malignancy [223]. Additionally, apoptotic bodies may also protect the circulating nucleic acids from enzymatic degradation [224].

Previously, the unusual anti-tumor role of TAM at the interface of cancer cell-derived apoptotic cells was mentioned [95]. It is known that phagocytes maintain tissue homeostasis by clearance of apoptotic cells. Such an effect action has also been described in conditioned medium from macrophages exposed to UV-killed cancer cells, which display TGFβ1-induced EMT inhibition, migration, and metastasis. Interestingly, apoptotic 344SQ (ApoSQ) cell-induced PPARγ activity in macrophages was shown to increase the exosomal PTEN levels, which was further taken up by recipient lung cancer cells. In syngeneic immunocompetent mice, a single injection of ApoSQ cells can inhibit lung metastasis as reflected in enhanced PPARγ/PTEN signaling both in tumor cells and in TAMs. Thus, the injection of apoptotic cancer cells can be used as an additional therapeutic option against cancer in addition to other strategies for identifying and targeting tumor-related dead cells (Fig. 2h).

## Conclusions

It is now well known that tumor cells can turn their surrounding niche into a hospitable home to better meet their growth needs and dissemination [3]. In response to hostile conditions such as oxygen deficiency, nutrients deficiency, accumulation of waste products, acidity, chemotherapy, etc. caused by the rapidly growing population of malignant cells, cancer cells can recruit their neighboring non-malignant cells including fibroblasts and immune cells as well as the non-cellular components for their own benefit [8, 10].

As discussed in this paper, tumor cells can make immune cells to suppress immune editing, or they can exploit CAFs/TAMs to elicit pro-inflammatory and proangiogenic states to favor cancer growth or consume them as energy sources if required. In addition, they can harness pericytes and TECs to promote angiogenesis. They can also orchestrate signaling pathways (e.g. EMT) to help them detach from their original residence to lead their way to other organs in the form of CTCs or promote signaling and horizontal transfer of genetic material through cfDNA, exosomes, and apoptotic bodies.

Understanding these interactions can help to implement better therapeutic regimes for cancer management, however, a combination of strategies appears to be more effective than single modalities, since the tumor heterogeneity arises from a variety of signaling pathways/cross talks existing in the network of communicating cancer cells. For example, in the view of metabolic plasticity [225, 226], therapies that target metabolism-modulating pathways would presumably be required to aim at parallel mechanisms accomplishing bio-energetic essentials, or to couple metabolic inhibitors with therapeutic interventions which conquer plasticity of cancer cells and hence the metabolic adaptation capacity. Since anti-tumor immunity suppression is progressively tied to key metabolic pathways’ activities and intratumoral metabolite levels in immune cells [227], the way by which CAF secretome could affect metabolism and immune cells function in the TME requires further investigation [228].

Importantly, cancer growth and in particular, metastatic expansion can be significantly reduced through targeting strategies to block and eliminate tumor-derived exosomes [187, 188] and/or aggressive CTCs [152, 154] from the circulation of cancer patients, as discussed in the earlier sections. In the same way, cfDNA, apoptotic bodies, and exosomes can be used as noninvasive biomarkers for early detection of cancer cells as well as predicting therapy success/relapse. However, due to their low concentrations in the bodily fluids, ongoing efforts are in the way to envision simple yet accurate point-of-care devices for efficient detection of these cancer cell-derived signatures in circulation.

Exosomes, on the other hand, have recently aroused great interest due to their potential as dual diagnostic and therapeutic tools. They provide valuable information regarding cancer cell secretome and signaling nodes/messages by which cells can communicate and even horizontally transfer genetic materials among each other. Plus, they can be engineered as natural vectors for ?A3B2 show $132#?>controlled drug release and gene therapy.

It is obvious that a complex procedure as smart and intelligent as the evolution and progression of cancer and its intense interaction with the surrounding environment needs to be explored in more detail, and that each topic addressed in this article could be expanded as a unique and comprehensive overview.

Together, we assume that disrupting tumor cell interactions can be exploited as a novel strategy for future cancer therapy regimes; however, further studies are still pending for clinical implementation in order to fully understand tumor cell interactions. This, in turn requires extensive research to test the potential and to outweigh the efficacy in suitable pre-clinical settings. Novel 3D systems and lab-on-chip devices can play an important role here, as they can be tailored to reconstruct almost any biological phenomenon/behavior of cancer cells at the cell-cell level up to an entire human body on a tiny chip that occurs in a more physiological environment comparable to the original tumor ecosystem.

## Data Availability

Not applicable.

## Abbreviations

ECM: Extracellular matrix
TME: Tumor microenvironment
MDR: Multi-drug resistance
CTCs: Circulating tumor cells
cfDNA: Cell-free DNA
HGT: Horizontal gene transfer
EMT: Epithelial-mesenchymal-transition
3D: Three dimensional
PDGF: Platelet-derived growth factor
VEGF: Vascular endothelial growth factor
2D: Two-dimensional
BBB: Blood-brain barrier
ECs: Endothelial cells
TECs: Tumor-derived endothelial cells
ALDH: Aldehyde dehydrogenase
TNFα: Tumor necrosis factor-alpha
CXCR: Chemokine receptors
CCR2: Chemokine (C-C motif) receptor 2
CAFs: Cancer-associated fibroblasts
α-SMA: Alpha-smooth muscle actin
MYL9: Myosin light chain 9
MYLK: Myosin light chain kinase
MMP2: Matrix metalloproteinase 2
DCN: Decorin
COL1A2: Collagen type I alpha 2
MCAM: Melanoma cell adhesion molecule
TAM: Tumor associated macrophage
MDSCs: Myeloid-derived suppressor cells
FGF: Fibroblast growth factor
TGFβ: Transforming growth factorβ
IL: Interleukin
LIF: Leukemia inhibitory factor
JAK/STAT: Janus kinase/signal transducers and activators of transcription
ILR1: Interleukin receptor, type I
TRACER: Tissue roll for the analysis of cellular environment and response
GPNMB: Glycoprotein nonmetastatic B
CCL: Chemokine ligand
CXCL: C-X-C motif chemokine
NADPH: Nicotinamide adenine dinucleotide phosphate
TCA: Tricarboxylic acid
M-CSF: Macrophage colony-stimulating factor
uPA: Urokinase plasminogen activator
ADM: Adrenomedullin
Sema4D: Semaphorin 4D
TP: Thymidine phosphorylase
NF-κB: Nuclear factor kappa -light-chain-enhancer of activated B cells
FN1: Fibronectin 1
TNC: Tenascin C
miRNAs: microRNAs
LOX: Lysyl oxidase
SPARC: Secreted protein acidic cysteine-rich
TIMP3: Tissue inhibitor of metalloproteinase 3
EpCAM: Epithelial cell adhesion molecule expression
CARS: Coherent anti-stokes raman scattering
(HER2): Human epidermal growth factor receptor 2
MVB-EVs: Multivesicular body-derived extracellular vesicles
TLR9: Toll-like receptor 9
MYD88: Myeloid differentiation primary response 88
BM: Bone marrow
cfCh: Cell-free chromatin
H2AX: H2A histone family member X
IFNγ: Interferon gamma
PD-L1: Programmed death-ligand 1
CTLA4: Cytotoxic T-lymphocyte-associated protein
CSF1R: Colony-stimulating factor 1 receptor
Treg: T regulatory
PFD: Pirfenidone
NPs: Nanoparticles
sTRAIL: Secretable TNF-related apoptosis inducing ligand
CHC: α-cyano-4-hydroxycinnamate
CQ: Chloroquine
Abs: Antibodies
BAPN: β-Aminopropionitrile
AAAs: Anti-angiogenic agents
VDAs: Vascular disrupting agents
CM: Conditioned media
VEGFR: VEGF-receptor
ATP: Adenosine triphosphate
DC: Dendritic cell
ACE: Angiotensin-converting enzyme
VWF: Von willebrand factor
PDCs: Peptide–drug conjugates

## References

[1] Jahanban-Esfahlan R, Seidi K, Monhemi H, Adli ADF, Minofar B, Zare P, Farajzadeh D, Farajnia S, Behzadi R, Abbasi MM (2017). RGD delivery of truncated coagulase to tumor vasculature affords local thrombotic activity to induce infarction of tumors in mice. Sci Rep.

[2] Jahanban-Esfahlan R, Seidi K, Banimohamad-Shotorbani B, Jahanban-Esfahlan A, Yousefi B (2017). Combination of nanotechnology with vascular targeting agents for effective cancer therapy. J Cell Physiol.

[3] Jahanban-Esfahlan R, Seidi K, Zarghami N (2017). Tumor vascular infarction: prospects and challenges. Int J Hematol.

[4] Hanahan D, Coussens LM (2012). Accessories to the crime: functions of cells recruited to the tumor microenvironment. Cancer Cell.

[5] Frisch J, Angenendt A, Hoth M, Prates Roma L, Lis AJC: STIM-Orai Channels and Reactive Oxygen Species in the Tumor Microenvironment 2019, 11:457. 10.3390/cancers11040457 PMC6520831 30935064

[6] Denisenko TV, Budkevich IN, Zhivotovsky BJCd, disease: Cell death-based treatment of lung adenocarcinoma. 2018, 9:117. 10.1038/s41419-017-0063-y PMC5833343 29371589

[7] Balkwill FR, Capasso M, Hagemann T: The tumor microenvironment at a glance. The Company of Biologists Ltd; 2012. 10.1242/jcs.116392 23420197

[8] Jahanban-Esfahlan R, de la Guardia M, Ahmadi D, Yousefi B (2017). Modulating tumor hypoxia by nanomedicine for effective cancer therapy. J Cell Physiol.

[9] Jahanban-Esfahlan R, Seidi K, Manjili MH, Jahanban-Esfahlan A, Javaheri T, Zare P (2019). Tumor cell dormancy: threat or opportunity in the fight against Cancer. Cancers.

[10] Seidi K, Neubauer HA, Moriggl R, Jahanban-Esfahlan R, Javaheri T (2018). Tumor target amplification: implications for nano drug delivery systems. J Control Release.

[11] Ungefroren H, Sebens S, Seidl D, Lehnert H, Hass R (2011). Interaction of tumor cells with the microenvironment. Cell CommunSignaling.

[12] Li W, Ng JM-K, Wong CC, Ng EKW, Yu JJO: Molecular alterations of cancer cell and tumour microenvironment in metastatic gastric cancer 2018:1. 10.1038/s41388-018-0341-x PMC6127089 29795331

[13] Tsao AS, Scagliotti GV, Bunn Jr PA, Carbone DP, Warren GW, Bai C, De Koning HJ, Yousaf-Khan AU, McWilliams A, Tsao MSJJoTO: Scientific advances in lung cancer 2015. 2016, 11:613–638. 10.1016/j.jtho.2016.03.012 27013409

[14] Cova TF, Bento DJ, Nunes SC (2019). Computational approaches in Theranostics: mining and predicting Cancer data. Pharmaceutics.

[15] Sounni NE, Noel A (2013). Targeting the tumor microenvironment for cancer therapy. Clin Chem.

[16] Oliver AJ, Lau PK, Unsworth AS, Loi S, Darcy PK, Kershaw MH, Slaney CYJFii: Tissue-dependent tumor microenvironments and their impact on immunotherapy responses. 2018;9:70. 10.3389/fimmu.2018.00070 PMC5797771 29445373

[17] Ayubi Joshagani MH, Dianat-Moghadam H, Seidi K, Jahanban-Esfahlan A, Zare P, Jahanban-Esfahlan R. Cell-free protein synthesis: the transition from batch reactions to minimal cells and microfluidic devices. Biotechnol Bioeng. 2019;117:1204–29. 10.1002/bit.27248 31840797

[18] Sleeboom JJF, Eslami Amirabadi H, Nair P, Sahlgren CM, den Toonder JMJ (2018). Metastasis in context: modeling the tumor microenvironment with cancer-on-a-chip approaches. Disease Models Mechanisms.

[19] Peela N, Truong D, Saini H, Chu H, Mashaghi S, Ham SL, Singh S, Tavana H, Mosadegh B, Nikkhah M (2017). Advanced biomaterials and microengineering technologies to recapitulate the stepwise process of cancer metastasis. Biomaterials.

[20] Kumar S, Weaver VM (2009). Mechanics, malignancy, and metastasis: the force journey of a tumor cell. Cancer Metastasis Rev.

[21] Friedl P, Wolf K (2003). Tumour-cell invasion and migration: diversity and escape mechanisms. Nat Rev Cancer.

[22] Kenny PA, Lee GY, Bissell MJ (2007). Targeting the tumor microenvironment. Front Biosci.

[23] Abbasi MM, Helli S, Monfaredan A, Jahanban-Esfahlan R (2015). Hesa-a improves clinical outcome of Oral carcinoma by affecting p53 gene expression in vivo. Asian Pac J Cancer Prev.

[24] Kloc M, Kubiak JZ, Li XC, Ghobrial RM (2015). Pericytes, microvasular dysfunction and chronic rejection. Transplantation.

[25] Baluk P, Morikawa S, Haskell A, Mancuso M, McDonald DM (2003). Abnormalities of basement membrane on blood vessels and endothelial sprouts in tumors. Am J Pathol.

[26] Birbrair A: Pericyte biology: development, homeostasis, and disease. In Pericyte Biology-Novel Concepts Springer; 2018: 1–3. 10.1007/978-3-030-02601-1_1 30523585

[27] Keskin D, Kim J, Cooke VG, Wu C-C, Sugimoto H, Gu C, De Palma M, Kalluri R, LeBleu VS (2015). Targeting vascular pericytes in hypoxic tumors increases lung metastasis via angiopoietin-2. Cell Rep.

[28] Hainsworth JD, Spigel DR, Sosman JA, Burris HA, Farley C, Cucullu H, Yost K, Hart LL, Sylvester L, Waterhouse DM (2007). Treatment of advanced renal cell carcinoma with the combination bevacizumab/erlotinib/imatinib: a phase I/II trial. Clinical Genitourinary Cancer.

[29] Nisancioglu MH, Betsholtz C, Genové G (2010). The absence of pericytes does not increase the sensitivity of tumor vasculature to vascular endothelial growth factor-a blockade. Cancer Res.

[30] Mezheyeuski A, Lindh MB, Guren TK, Dragomir A, Pfeiffer P, Kure EH, Ikdahl T, Skovlund E, Corvigno S, Strell C (2016). Survival-associated heterogeneity of marker-defined perivascular cells in colorectal cancer. Oncotarget.

[31] Xian X, Håkansson J, Ståhlberg A, Lindblom P, Betsholtz C, Gerhardt H, Semb H (2006). Pericytes limit tumor cell metastasis. J Clin Invest.

[32] Yonenaga Y, Mori A, Onodera H, Yasuda S, Oe H, Fujimoto A, Tachibana T, Imamura M (2005). Absence of smooth muscle actin-positive pericyte coverage of tumor vessels correlates with hematogenous metastasis and prognosis of colorectal cancer patients. Oncology.

[33] Hong J, Tobin NP, Rundqvist H, Li T, Lavergne M, García-Ibáñez Y, Qin H, Paulsson J, Zeitelhofer M, Adzemovic MZ (2015). Role of tumor pericytes in the recruitment of myeloid-derived suppressor cells. J National Cancer Institute.

[34] Cooke VG, LeBleu VS, Keskin D, Khan Z, O'Connell JT, Teng Y, Duncan MB, Xie L, Maeda G, Vong S (2012). Pericyte depletion results in hypoxia-associated epithelial-to-mesenchymal transition and metastasis mediated by met signaling pathway. Cancer Cell.

[35] Cheng L, Huang Z, Zhou W, Wu Q, Donnola S, Liu JK, Fang X, Sloan AE, Mao Y, Lathia JD (2013). Glioblastoma stem cells generate vascular pericytes to support vessel function and tumor growth. Cell.

[36] Murgai M, Ju W, Eason M, Kline J, Beury DW, Kaczanowska S, Miettinen MM, Kruhlak M, Lei H, Shern JF (2017). KLF4-dependent perivascular cell plasticity mediates pre-metastatic niche formation and metastasis. Nat Med.

[37] Campisi M, Shin Y, Osaki T, Hajal C, Chiono V, Kamm RD (2018). 3D self-organized microvascular model of the human blood-brain barrier with endothelial cells, pericytes and astrocytes. Biomaterials.

[38] Wang X, Sun Q, Pei J (2018). Microfluidic-based 3D engineered microvascular networks and their applications in vascularized Microtumor models. Micromachines.

[39] Zhao H, Chappell JC (2019). Microvascular bioengineering: a focus on pericytes. J Biol Eng.

[40] Salazar N, Zabel BA. Support of tumor endothelial cells by chemokine receptors. Front Immunol. 2019;10. 10.3389/fimmu.2019.00147 PMC6375834 30800123

[41] Dudley AC (2012). Tumor endothelial cells. Cold Spring Harbor perspectives in medicine.

[42] Aird WC (2009). Molecular heterogeneity of tumor endothelium. Cell Tissue Res.

[43] Akiyama K, Ohga N, Hida Y, Kawamoto T, Sadamoto Y, Ishikawa S, Maishi N, Akino T, Kondoh M, Matsuda A (2012). Tumor endothelial cells acquire drug resistance by MDR1 up-regulation via VEGF signaling in tumor microenvironment. Am J Pathol.

[44] Hida K, Maishi N, Akiyama K, Ohmura-Kakutani H, Torii C, Ohga N, Osawa T, Kikuchi H, Morimoto H, Morimoto M (2017). Tumor endothelial cells with high aldehyde dehydrogenase activity show drug resistance. Cancer Sci.

[45] Dianat-Moghadam H, Heydarifard M, Jahanban-Esfahlan R, Panahi Y, Hamishehkar H, Pouremamali F, Rahbarghazi R, Nouri M (2018). Cancer stem cells-emanated therapy resistance: implications for liposomal drug delivery systems. J Control Release.

[46] Abdalla AME, Xiao L, Ullah MW, Yu M, Ouyang C, Yang G (2018). Current challenges of Cancer anti-angiogenic therapy and the promise of Nanotherapeutics. Theranostics.

[47] Abbasi MM, Mehdipour M, Monfaredan A, Jahanban-Esfahlan R (2015). Hesa-a Down-regulates erb/b2 oncogene expression and improves outcome of Oral carcinoma in a rat model. Asian Pac J Cancer Prev.

[48] Charles N, Ozawa T, Squatrito M, Bleau AM, Brennan CW, Hambardzumyan D, Holland EC (2010). Perivascular nitric oxide activates notch signaling and promotes stem-like character in PDGF-induced glioma cells. Cell Stem Cell.

[49] Jeon HM, Kim SH, Jin X, Park JB, Kim SH, Joshi K, Nakano I, Kim H (2014). Crosstalk between glioma-initiating cells and endothelial cells drives tumor progression. Cancer Res.

[50] Daei Farshchi Adli A, Jahanban-Esfahlan R, Seidi K, Samandari-Rad S, Zarghami N. An overview on vadimezan (dmxaa), the vascular disrupting agent. Chem Biol Drug Des. 2017;91(5):996–1006. 10.1111/cbdd.13166 29288534

[51] Seidi K, Jahanban-Esfahlan R, Zarghami N (2017). Tumor rim cells: from resistance to vascular targeting agents to complete tumor ablation. Tumour Biol.

[52] Zhang Y, Xiong X, Huai Y, Dey A, Hossen MN, Roy RV, Elechalawar CK, Rao G, Bhattacharya R, Mukherjee P (2019). Gold nanoparticles disrupt tumor microenvironment - endothelial cell cross talk to inhibit Angiogenic phenotypes in vitro. Bioconjug Chem.

[53] Missiaen R, Morales-Rodriguez F, Eelen G, Carmeliet P (2017). Targeting endothelial metabolism for anti-angiogenesis therapy: a pharmacological perspective. Vasc Pharmacol.

[54] Nomura T, Yamakawa M, Shimaoka T, Hirai T, Koizumi N, Maruyama K, Utoguchi N (2019). Development of dendritic cell-based immunotherapy targeting tumor blood vessels in a mouse model of lung metastasis. Biol Pharm Bull.

[55] Shoval H, Karsch-Bluman A, Brill-Karniely Y, Stern T, Zamir G, Hubert A, Benny O (2017). Tumor cells and their crosstalk with endothelial cells in 3D spheroids. Sci Rep.

[56] Zervantonakis IK, Hughes-Alford SK, Charest JL, Condeelis JS, Gertler FB, Kamm RD (2012). Three-dimensional microfluidic model for tumor cell intravasation and endothelial barrier function. Proc Natl Acad Sci.

[57] Jeon JS, Bersini S, Gilardi M, Dubini G, Charest JL, Moretti M, Kamm RD (2015). Human 3D vascularized organotypic microfluidic assays to study breast cancer cell extravasation. Proc Natl Acad Sci U S A.

[58] Lee SW, Kwak HS, Kang M-H, Park Y-Y, Jeong GSJSr. Fibroblast-associated tumour microenvironment induces vascular structure-networked tumouroid. Sci Rep. 2018;8:2365. 10.1038/s41598-018-20886-0 PMC5799156 29403007

[59] Kalluri RJNRC. The biology and function of fibroblasts in cancer. Nat Rev Cancer. 2016;16:582–98. 10.1038/nrc.2016.73 27550820

[60] Liu T, Zhou L, Li D, Andl T, Zhang Y. Cancer-associated fibroblasts build and secure the tumor microenvironment. Front Cell Dev Biol. 2019;7:60. 10.3389/fcell.2019.00060 PMC6492564 31106200

[61] Nurmik M, Ullmann P, Rodriguez F, Haan S, Letellier E (2020). In search of definitions: Cancer-associated fibroblasts and their markers. Int J Cancer.

[62] Nishishita R, Morohashi S, Seino H, Wu Y, Yoshizawa T, Haga T, Saito K, Hakamada K, Fukuda S, Kijima H (2018). Expression of cancer-associated fibroblast markers in advanced colorectal cancer. Oncol Lett.

[63] Brunel A, Samain R, Neuzillet C. Bousquet CJTCR: identification of two cancer-associated fibroblast markers revealing stromal heterogeneity in sustaining cancer progression and chemoresistance. Trans Cancer Res. 2018:S718–21.

[64] Monteran L, Erez N (2019). The dark side of fibroblasts: Cancer-associated fibroblasts as mediators of immunosuppression in the tumor microenvironment. Front Immunol.

[65] Liu T, Han C, Wang S, Fang P, Ma Z, Xu L, Yin R (2019). Cancer-associated fibroblasts: an emerging target of anti-cancer immunotherapy. J Hematol Oncol.

[66] Gok Yavuz B, Gunaydin G, Gedik ME, Kosemehmetoglu K, Karakoc D, Ozgur F, Guc D (2019). Cancer associated fibroblasts sculpt tumour microenvironment by recruiting monocytes and inducing immunosuppressive PD-1+ TAMs. Sci Rep.

[67] Wang F-T, Sun W, Zhang J-T, Fan Y-Z (2019). Cancer-associated fibroblast regulation of tumor neo-angiogenesis as a therapeutic target in cancer. Oncol Lett.

[68] Tang D, Gao J, Wang S, Ye N, Chong Y, Huang Y, Wang J, Li B, Yin W, Wang D (2016). Cancer-associated fibroblasts promote angiogenesis in gastric cancer through galectin-1 expression. Tumour Biol.

[69] Zhou W, Xu G, Wang Y, Xu Z, Liu X, Xu X, Ren G, Tian K (2017). Oxidative stress induced autophagy in cancer associated fibroblast enhances proliferation and metabolism of colorectal cancer cells. Cell Cycle.

[70] Lisanti MP, Martinez-Outschoorn UE, Chiavarina B, Pavlides S, Whitaker-Menezes D, Tsirigos A, Witkiewicz AK, Lin Z, Balliet RM, Howell A (2010). Understanding the" lethal" drivers of tumor-stroma co-evolution: emerging role (s) for hypoxia, oxidative stress and autophagy/mitophagy in the tumor microenvironment. Cancer Biol Therapy.

[71] Yan Y, Chen X, Wang X, Zhao Z, Hu W, Zeng S, Wei J, Yang X, Qian L, Zhou S (2019). The effects and the mechanisms of autophagy on the cancer-associated fibroblasts in cancer. J Exp Clin Cancer Res.

[72] Curtis M, Kenny HA, Ashcroft B, Mukherjee A, Johnson A, Zhang Y, Helou Y, Batlle R, Liu X, Gutierrez N: Fibroblasts mobilize tumor cell glycogen to promote proliferation and metastasis. Cell Metabolism 2019, 29:141–155. e149. 10.1016/j.cmet.2018.08.007 PMC6326875 30174305

[73] Biffi G, Oni TE, Spielman B, Hao Y, Elyada E, Park Y, Preall J, Tuveson DA. IL-1-induced JAK/STAT signaling is antagonized by TGF-beta to shape CAF heterogeneity in pancreatic ductal adenocarcinoma. Cancer Discov. 2018:9:282–301. 10.1158/2159-8290.CD-18-0710 PMC6368881 30366930

[74] Young M, Rodenhizer D, Dean T, D'Arcangelo E, Xu B, Ailles L, McGuigan AP (2018). A TRACER 3D co-culture tumour model for head and neck cancer. Biomaterials.

[75] Truong DD, Kratz A, Park JG, Barrientos ES, Saini H, Nguyen T, Pockaj B, Mouneimne G, LaBaer J, Nikkhah M. A human Organotypic microfluidic tumor model permits investigation of the interplay between patient-derived fibroblasts and breast Cancer cells. Cancer Res. 2019;79:3139–51. 10.1158/0008-5472.CAN-18-2293 PMC6664809 30992322

[76] Kraman M, Bambrough PJ, Arnold JN, Roberts EW, Magiera L, Jones JO, Gopinathan A, Tuveson DA, Fearon DT (2010). Suppression of antitumor immunity by stromal cells expressing fibroblast activation protein–α. Science.

[77] Quail DF, Joyce JA (2013). Microenvironmental regulation of tumor progression and metastasis. Nat Med.

[78] Mercier I, Camacho J, Titchen K, Gonzales DM, Quann K, Bryant KG, Molchansky A, Milliman JN, Whitaker-Menezes D, Sotgia F (2012). Caveolin-1 and accelerated host aging in the breast tumor microenvironment: chemoprevention with rapamycin, an mTOR inhibitor and anti-aging drug. Am J Pathol.

[79] Ernsting MJ, Hoang B, Lohse I, Undzys E, Cao P, Do T, Gill B, Pintilie M, Hedley D, Li S-D (2015). Targeting of metastasis-promoting tumor-associated fibroblasts and modulation of pancreatic tumor-associated stroma with a carboxymethylcellulose-docetaxel nanoparticle. J Control Release.

[80] Sherman MH, Ruth TY, Engle DD, Ding N, Atkins AR, Tiriac H, Collisson EA, Connor F, Van Dyke T, Kozlov S (2014). Vitamin D receptor-mediated stromal reprogramming suppresses pancreatitis and enhances pancreatic cancer therapy. Cell.

[81] Zhang J, Miao L, Guo S, Zhang Y, Zhang L, Satterlee A, Kim WY, Huang L (2014). Synergistic anti-tumor effects of combined gemcitabine and cisplatin nanoparticles in a stroma-rich bladder carcinoma model. J Control Release.

[82] Nunes AS, Barros AS, Costa EC, Moreira AF, Correia IJJB. Bioengineering: 3D tumor spheroids as in vitro models to mimic in vivo human solid tumors resistance to therapeutic drugs. Biotechnol Bioeng. 2019;116:206–26. 10.1002/bit.26845 30367820

[83] Miao L, Liu Q, Lin CM, Luo C, Wang Y, Liu L, Yin W, Hu S, Kim WY, Huang L (2017). Targeting tumor-associated fibroblasts for therapeutic delivery in desmoplastic tumors. Cancer Res.

[84] Takai K, Le A, Weaver VM, Werb Z (2016). Targeting the cancer-associated fibroblasts as a treatment in triple-negative breast cancer. Oncotarget.

[85] Li X, Huang F, Xu X, Hu S (2018). Polyclonal rabbit anti-Cancer-associated fibroblasts globulins induce Cancer cells apoptosis and inhibit tumor growth. Int J Biol Sci.

[86] Zhang X, Schönrogge M, Eichberg J, Wendt EHU, Kumstel S, Stenzel J, Lindner T, Jaster R, Krause BJ, Vollmar BJFio. Blocking autophagy in cancer-associated fibroblasts supports chemotherapy of pancreatic cancer cells. Front Oncol. 2018;8:590. 10.3389/fonc.2018.00590 PMC6290725 30568920

[87] Zhang R, Qi F, Zhao F, Li G, Shao S, Zhang X, Yuan L, Feng Y (2019). Cancer-associated fibroblasts enhance tumor-associated macrophages enrichment and suppress NK cells function in colorectal cancer. Cell Death Dis.

[88] Noy R, Pollard JW (2014). Tumor-associated macrophages: from mechanisms to therapy. Immunity.

[89] Larionova I, Cherdyntseva N, Liu T, Patysheva M, Rakina M, Kzhyshkowska J (2019). Interaction of tumor-associated macrophages and cancer chemotherapy. OncoImmunol.

[90] Laviron M, Boissonnas A. Ontogeny of tumor-associated macrophages. Front Immunol. 2019;10:1799. 10.3389/fimmu.2019.01799 PMC6684758 31417566

[91] Mantovani A, Sica A, Sozzani S, Allavena P, Vecchi A, Locati M (2004). The chemokine system in diverse forms of macrophage activation and polarization. Trends Immunol.

[92] Cassetta L, Kitamura T (2018). Targeting tumor-associated macrophages as a potential strategy to enhance the response to immune checkpoint inhibitors. Front Cell Developmental Biol.

[93] Solinas G, Schiarea S, Liguori M, Fabbri M, Pesce S, Zammataro L, Pasqualini F, Nebuloni M, Chiabrando C, Mantovani A (2010). Tumor-conditioned macrophages secrete migration-stimulating factor: a new marker for M2-polarization, influencing tumor cell motility. J Immunol.

[94] Wei C, Yang C, Wang S, Shi D, Zhang C, Lin X, Liu Q, Dou R, Xiong B (2019). Crosstalk between cancer cells and tumor associated macrophages is required for mesenchymal circulating tumor cell-mediated colorectal cancer metastasis. Mol Cancer.

[95] Kim Y-B, Ahn Y-H, Jung J-H, Lee Y-J, Lee J-H, Kang JL. Programming of macrophages by UV-irradiated apoptotic cancer cells inhibits cancer progression and lung metastasis. Cell Mol Immunol. 2019;16:851–67. 10.1038/s41423-019-0209-1 PMC6828747 30842627

[96] Lin Y, Xu J, Lan H (2019). Tumor-associated macrophages in tumor metastasis: biological roles and clinical therapeutic applications. J Hematol Oncol.

[97] Qian B, Deng Y, Im JH, Muschel RJ, Zou Y, Li J, Lang RA, Pollard JW (2009). A distinct macrophage population mediates metastatic breast cancer cell extravasation, establishment and growth. PLoS One.

[98] Udeabor SE, Adisa AO, Orlowska A, Sader RA, Ghanaati S (2017). Tumor-associated macrophages, angiogenesis, and tumor cell migration in oral squamous cell carcinoma. Ann Afr Med.

[99] Chen Y, Song Y, Du W, Gong L, Chang H, Zou Z (2019). Tumor-associated macrophages: an accomplice in solid tumor progression. J Biomed Sci.

[100] Chen Y, Tan W, Wang C (2018). Tumor-associated macrophage-derived cytokines enhance cancer stem-like characteristics through epithelial-mesenchymal transition. Onco Targets Ther.

[101] Kowal J, Kornete M, Joyce JA (2019). Re-education of macrophages as a therapeutic strategy in cancer. Immunotherapy.

[102] Zhan X, Jia L, Niu Y, Qi H, Chen X, Zhang Q, Zhang J, Wang Y, Dong L, Wang C (2014). Targeted depletion of tumour-associated macrophages by an alendronate-glucomannan conjugate for cancer immunotherapy. Biomaterials.

[103] Mantovani A, Marchesi F, Malesci A, Laghi L, Allavena P (2017). Tumour-associated macrophages as treatment targets in oncology. Nat Rev Clin Oncol.

[104] Kudo M (2019). Combination Cancer immunotherapy with molecular targeted agents/anti-CTLA-4 antibody for hepatocellular carcinoma. Liver Cancer.

[105] DeNardo DG, Brennan DJ, Rexhepaj E, Ruffell B, Shiao SL, Madden SF, Gallagher WM, Wadhwani N, Keil SD, Junaid SA (2011). Leukocyte complexity predicts breast cancer survival and functionally regulates response to chemotherapy. Cancer Discovery.

[106] Pyonteck SM, Akkari L, Schuhmacher AJ, Bowman RL, Sevenich L, Quail DF, Olson OC, Quick ML, Huse JT, Teijeiro V (2013). CSF-1R inhibition alters macrophage polarization and blocks glioma progression. Nat Med.

[107] Ries CH, Cannarile MA, Hoves S, Benz J, Wartha K, Runza V, Rey-Giraud F, Pradel LP, Feuerhake F, Klaman I (2014). Targeting tumor-associated macrophages with anti-CSF-1R antibody reveals a strategy for cancer therapy. Cancer Cell.

[108] Arlauckas SP, Garris CS, Kohler RH, Kitaoka M, Cuccarese MF, Yang KS, Miller MA, Carlson JC, Freeman GJ, Anthony RMJStm. In vivo imaging reveals a tumor-associated macrophage–mediated resistance pathway in anti–PD-1 therapy. Sci Transl Med. 2017;9:eaal3604. 10.1126/scitranslmed.aal3604 PMC5734617 28490665

[109] de Taeye SW, Rispens T, Vidarsson G (2019). The ligands for human IgG and their effector functions. Antibodies.

[110] Li R, Hebert JD, Lee TA, Xing H, Boussommier-Calleja A, Hynes RO, Lauffenburger DA, Kamm RD. Macrophage-secreted TNFα and TGFβ1 Influence Migration Speed and Persistence of Cancer Cells in 3D Tissue Culture via Independent Pathways. Cancer Res. 2017;77:279–20. 10.1158/0008-5472.CAN-16-0442 PMC5243269 27872091

[111] Han J, Zhen J, Go G, Choi Y, Ko SY, Park J-O (2016). Park SJSr: hybrid-actuating macrophage-based microrobots for active cancer therapy. Sci Rep.

[112] Jahanban-Esfahlan A, Seidi K, Jaymand M, Schmidt TL, Zare P, Javaheri T, Jahanban-Esfahlan R. Dynamic DNA nanostructures in biomedicine: beauty, utility and limits. J Control Release. 2019;315:166–85. 10.1016/j.jconrel.2019.10.003 31669209

[113] Frantz C, Stewart KM, Weaver VM (2010). The extracellular matrix at a glance. J Cell Sci.

[114] Hynes RO (2009). The extracellular matrix: not just pretty fibrils. Science.

[115] Lu P, Takai K, Weaver VM, Werb Z (2011). Extracellular matrix degradation and remodeling in development and disease. Cold Spring Harb Perspect Biol.

[116] Theocharis AD, Skandalis SS, Gialeli C, Karamanos NK (2016). Extracellular matrix structure. Adv Drug Deliv Rev.

[117] Walker C, Mojares E, del Río HA (2018). Role of extracellular matrix in development and cancer progression. Int J Mol Sci.

[118] Lu P, Weaver VM, Werb Z (2012). The extracellular matrix: a dynamic niche in cancer progression. J Cell Biol.

[119] Kim S-H, Turnbull J, Guimond S (2011). Extracellular matrix and cell signalling: the dynamic cooperation of integrin, proteoglycan and growth factor receptor. J Endocrinol.

[120] Eble JA, Niland S (2019). The extracellular matrix in tumor progression and metastasis. Clin Exper Metastasis.

[121] Poltavets V, Kochetkova M, Pitson SM, Samuel MS. The role of the extracellular matrix and its molecular and cellular regulators in Cancer cell plasticity. Front Oncol. 2018;8:431. 10.3389/fonc.2018.00431 PMC6189298 30356678

[122] Jabłońska-Trypuć A, Matejczyk M, Rosochacki S (2016). Matrix metalloproteinases (MMPs), the main extracellular matrix (ECM) enzymes in collagen degradation, as a target for anticancer drugs. J Enzyme Inhibition Med Chem.

[123] Zhang R, Ma M, Lin X-H, Liu H-H, Chen J, Chen J, Gao D-M, Cui J-F, Ren Z-G, Chen R-X (2018). Extracellular matrix collagen I promotes the tumor progression of residual hepatocellular carcinoma after heat treatment. BMC Cancer.

[124] Xu S, Xu H, Wang W, Li S, Li H, Li T, Zhang W, Yu X, Liu L (2019). The role of collagen in cancer: from bench to bedside. J Transl Med.

[125] Naito Y, Sakamoto N, Oue N, Yashiro M, Sentani K, Yanagihara K, Hirakawa K, Yasui W (2014). MicroRNA-143 regulates collagen type III expression in stromal fibroblasts of scirrhous type gastric cancer. Cancer Sci.

[126] Mu W, Rana S, Zoller M (2013). Host matrix modulation by tumor exosomes promotes motility and invasiveness. Neoplasia.

[127] Natarajan S, Foreman KM, Soriano MI, Rossen NS, Shehade H, Fregoso DR, Eggold JT, Krishnan V, Dorigo O, Krieg AJ. Collagen remodeling in the hypoxic tumor-mesothelial niche promotes ovarian cancer metastasis. Cancer Res. 2019. 10.1158/0008-5472.CAN-18-2616. 10.1158/0008-5472.CAN-18-2616 PMC6822898 30862717

[128] Saini H, Eliato K, Silva C, Allam M, Mouneimne G, Ros R, Nikkhah M. The role of Desmoplasia and stromal fibroblasts on anti-cancer drug resistance in a microengineered tumor model. Cell Mol Bioeng. 2018;11:419–33. 10.1007/s12195-018-0544-9 PMC6816733 31719892

[129] Provenzano PP, Eliceiri KW, Campbell JM, Inman DR, White JG, Keely PJ (2006). Collagen reorganization at the tumor-stromal interface facilitates local invasion. BMC Med.

[130] Hajdú I, Kardos J, Major B, Fabó G, Lőrincz Z, Cseh S, Dormán G (2018). Inhibition of the LOX enzyme family members with old and new ligands. Selectivity analysis revisited. Bioorg Med Chem Lett.

[131] Puente A, Fortea JI, Cabezas J, Arias Loste MT, Iruzubieta P, Llerena S, Huelin P, Fábrega E, Crespo J (2019). LOXL2—a new target in Antifibrogenic therapy?. Int J Mol Sci.

[132] Raavé R, van Kuppevelt TH, Daamen WF (2018). Chemotherapeutic drug delivery by tumoral extracellular matrix targeting. J Control Release.

[133] Orend G, Chiquet-Ehrismann R (2006). Tenascin-C induced signaling in cancer. Cancer Lett.

[134] Lowy CM, Oskarsson T (2015). Tenascin C in metastasis: a view from the invasive front. Cell Adhes Migr.

[135] Dal Corso A, Gébleux R, Murer P, Soltermann A, Neri D (2017). A non-internalizing antibody-drug conjugate based on an anthracycline payload displays potent therapeutic activity in vivo. J Control Release.

[136] Chen B, Dai W, Mei D, Liu T, Li S, He B, He B, Yuan L, Zhang H, Wang X (2016). Comprehensively priming the tumor microenvironment by cancer-associated fibroblast-targeted liposomes for combined therapy with cancer cell-targeted chemotherapeutic drug delivery system. J Control Release.

[137] Ishihara J, Ishihara A, Sasaki K, Lee SS-Y, Williford J-M, Yasui M, Abe H, Potin L, Hosseinchi P, Fukunaga K (2019). Targeted antibody and cytokine cancer immunotherapies through collagen affinity. Sci Transl Med.

[138] Park J, Kim S, Saw PE, Lee IH, Yu MK, Kim M, Lee K, Kim YC, Jeong YY, Jon S (2012). Fibronectin extra domain B-specific aptide conjugated nanoparticles for targeted cancer imaging. J Control Release.

[139] Okur AC, Erkoc P, Kizilel S (2016). Targeting cancer cells via tumor-homing peptide CREKA functional PEG nanoparticles. Colloids Surf B Biointerfaces.

[140] Upreti M, Jyoti A, Johnson SE, Swindell EP, Napier D, Sethi P, Chan R, Feddock JM, Weiss HL, O'Halloran TV, Evers BM (2016). Radiation-enhanced therapeutic targeting of galectin-1 enriched malignant stroma in triple negative breast cancer. Oncotarget.

[141] Miot-Noirault E, Vidal A, Morlieras J, Bonazza P, Auzeloux P, Besse S, Dauplat MM, Peyrode C, Degoul F, Billotey C (2014). Small rigid platforms functionalization with quaternary ammonium: targeting extracellular matrix of chondrosarcoma. Nanomedicine.

[142] Jayatilaka H, Tyle P, Chen JJ, Kwak M, Ju J, Kim HJ, Lee JS, Wu P-H, Gilkes DM, Fan R (2017). Synergistic IL-6 and IL-8 paracrine signalling pathway infers a strategy to inhibit tumour cell migration. Nat Commun.

[143] Krol I, Castro-Giner F, Maurer M, Gkountela S, Szczerba BM, Scherrer R, Coleman N, Carreira S, Bachmann F, Anderson SJBjoc. Detection of circulating tumour cell clusters in human glioblastoma.Br J Cancer. 2018;119:487–91. 10.1038/s41416-018-0186-7 PMC6134152 30065256

[144] Shishido SN, Carlsson A, Nieva J, Bethel K, Hicks JB, Bazhenova L, Kuhn P (2019). Circulating tumor cells as a response monitor in stage IV non-small cell lung cancer. J Transl Med.

[145] Yap Y-S, Leong MC, Chua YW, Loh KWJ, Lee GE, Lim EH, Dent R, Ng RCH, Lim JH-C, Singh G (2019). Detection and prognostic relevance of circulating tumour cells (CTCs) in Asian breast cancers using a label-free microfluidic platform. PLoS One.

[146] Adams DL, Adams DK, Stefansson S, Haudenschild C, Martin SS, Charpentier M, Chumsri S, Cristofanilli M, Tang C-M, Alpaugh RK (2016). Mitosis in circulating tumor cells stratifies highly aggressive breast carcinomas. Breast Cancer Res.

[147] Kim M-Y, Oskarsson T, Acharyya S, Nguyen DX, Zhang XH-F, Norton L, Massagué J (2009). Tumor self-seeding by circulating cancer cells. Cell.

[148] Jayatilaka H, Phillip JM. Targeting metastasis through the inhibition of interleukin 6 and 8. Future Medicine. 2019. 10.2217/bmt-2019-0002.

[149] Martín M, Custodio S, de las Casas M-LM, García-Sáenz J-Á, de la Torre J-C, Bellón-Cano J-M, López-Tarruella S, Vidaurreta-Lazaro M, de la Orden V, Jerez YJTo. Circulating tumor cells following first chemotherapy cycle: an early and strong predictor of outcome in patients with metastatic breast cancer. Oncologist. 2013;18:917–23. 10.1634/theoncologist.2012-0479 PMC3755928 23873719

[150] Rack B, Schindlbeck C, Juckstock J, Andergassen U, Hepp P, Zwingers T, Friedl TW, Lorenz R, Tesch H, Fasching PA, et al. Circulating tumor cells predict survival in early average-to-high risk breast cancer patients. J Natl Cancer Inst. 2014;106:dju066. 10.1093/jnci/dju066 PMC4112925 24832787

[151] Yan W-T, Cui X, Chen Q, Li Y-F, Cui Y-H, Wang Y, Jiang JJSr. Circulating tumor cell status monitors the treatment responses in breast cancer patients: a meta-analysis . Sci Rep. 2017;7:43464. 10.1038/srep43464 PMC5364512 28337998

[152] Kim YR, Yoo JK, Jeong CW, Choi JW (2018). Selective killing of circulating tumor cells prevents metastasis and extends survival. J Hematol Oncol.

[153] Lian S, Xie R, Ye Y, Lu Y, Cheng Y, Xie X, Li S, Jia LJSr. Dual blockage of both PD-L1 and CD47 enhances immunotherapy against circulating tumor cells. Sci Rep. 2019;9:4532. 10.1038/s41598-019-40241-1 PMC6418176 30872703

[154] Dong H, Han L, Wu Z-S, Zhang T, Xie J, Ma J, Wang J, Li T, Gao Y, Shao J (2017). Biostable aptamer rings conjugated for targeting two biomarkers on circulating tumor cells in vivo with great precision. Chem Mater.

[155] Jahanban-Esfahlan R, Seidi K, Jahanban-Esfahlan A, Jaymand M, Alizadeh E, Majdi H, Najjar R, Javaheri T, Zare P. Static DNA nanostructures for cancer theranostics: Recent progress in design and applications. Nannotechnol Sci Appl. 2019;12:25–46. 10.2147/NSA.S227193 PMC6800557 31686793

[156] Song P, Ye D, Zuo X, Li J, Wang J, Liu H, Hwang MT, Chao J, Su S, Wang L (2017). DNA hydrogel with Aptamer-toehold-based recognition, cloaking, and Decloaking of circulating tumor cells for live cell analysis. Nano Lett.

[157] Straume O, Akslen L (2005). Strong expression of ID1 protein is associated with decreased survival, increased expression of ephrin-A1/EPHA2, and reduced thrombospondin-1 in malignant melanoma. Br J Cancer.

[158] Thaker PH, Deavers M, Celestino J, Thornton A, Fletcher MS, Landen CN, Kinch MS, Kiener PA, Sood AK (2004). EphA2 expression is associated with aggressive features in ovarian carcinoma. Clin Cancer Res.

[159] Han L, Dong Z, Qiao Y, Kristensen GB, Holm R, Nesland JM, Suo Z (2005). The clinical significance of EphA2 and Ephrin A-1 in epithelial ovarian carcinomas. Gynecol Oncol.

[160] Walker-Daniels J, Coffman K, Azimi M, Rhim J, Bostwick D, Snyder P, Kerns B, Waters D, Kinch M (1999). Overexpression of the EphA2 tyrosine kinase in prostate cancer. Prostate.

[161] Chen P, Huang Y, Zhang B, Wang Q, Bai P (2014). EphA2 enhances the proliferation and invasion ability of LNCaP prostate cancer cells. Oncol Lett.

[162] Kinch MS, Moore M-B, Harpole DH (2003). Predictive value of the EphA2 receptor tyrosine kinase in lung cancer recurrence and survival. Clin Cancer Res.

[163] Song W, Ma Y, Wang J, Brantley-Sieders D, Chen J (2014). JNK Signaling mediates EPHA2-dependent tumor cell proliferation, motility, and Cancer stem cell–like properties in non–small cell lung Cancer. Cancer Res.

[164] Brantley-Sieders DM, Jiang A, Sarma K, Badu-Nkansah A, Walter DL, Shyr Y, Chen J (2011). Eph/ephrin profiling in human breast cancer reveals significant associations between expression level and clinical outcome. PLoS One.

[165] Chukkapalli S, Amessou M, Dilly AK, Dekhil H, Zhao J, Liu Q, Bejna A, Thomas RD, Bandyopadhyay S, Bismar TA (2014). Role of the EphB2 receptor in autophagy, apoptosis and invasion in human breast cancer cells. Exp Cell Res.

[166] Wang S, Placzek WJ, Stebbins JL, Mitra S, Noberini R, Koolpe M, Zhang Z, Dahl R, Pasquale EB, Pellecchia M (2012). Novel targeted system to deliver chemotherapeutic drugs to EphA2-expressing cancer cells. J Med Chem.

[167] Quinn BA, Wang S, Barile E, Das SK, Emdad L, Sarkar D, De SK, Kharagh SM, Stebbins JL, Pandol SJ (2016). Therapy of pancreatic cancer via an EphA2 receptor-targeted delivery of gemcitabine. Oncotarget.

[168] Salem AF, Wang S, Billet S, Chen J-F, Udompholkul P, Gambini L, Baggio C, Tseng H-R, Posadas EM, Bhowmick NA, Pellecchia M (2018). Reduction of circulating Cancer cells and metastases in breast-Cancer models by a potent EphA2-agonistic peptide–drug conjugate. J Med Chem.

[169] Valcz G, Galamb O, Krenács T, Spisák S, Kalmár A, Patai ÁV, Wichmann B, Dede K, Tulassay Z, Molnár BJMP. Exosomes in colorectal carcinoma formation: ALIX under the magnifying glass. Modern Pathology. 2016;29:928. 10.1038/modpathol.2016.72 27150162

[170] Takahashi A, Okada R, Nagao K, Kawamata Y, Hanyu A, Yoshimoto S, Takasugi M, Watanabe S, Kanemaki MT, Obuse CJNc. Exosomes maintain cellular homeostasis by excreting harmful DNA from cells.Nat Commun. 2017;8:15287. 10.1038/ncomms15287 PMC5440838 28508895

[171] Németh A, Orgovan N, Sódar BW, Osteikoetxea X, Pálóczi K, Szabó-Taylor KÉ, Vukman KV, Kittel Á, Turiák L, Wiener ZJSr. Antibiotic-induced release of small extracellular vesicles (exosomes) with surface-associated DNA. Sci Rep. 2017;7:8202. 10.1038/s41598-017-08392-1 PMC5557920 28811610

[172] Valcz G, Buzás EI, Szállási Z, Kalmár A, Krenács T, Tulassay Z, Igaz P. Molnár BJNbc. Perspective: bidirectional exosomal transport between cancer stem cells and their fibroblast-rich microenvironment during metastasis formation. 2018;4:18. 10.1038/s41523-018-0071-9 PMC6048124 30038960

[173] Sullivan R, Maresh G, Zhang X, Salomon C, Hooper J, Margolin D, Li L (2017). The emerging roles of extracellular vesicles as communication vehicles within the tumor microenvironment and beyond. Front Endocrinol (Lausanne).

[174] Wendler F, Stamp GW, Giamas G (2016). Tumor–stromal cell communication: small vesicles signal big changes. Trends Cancer.

[175] Yu Y, Abudula M, Li C, Chen Z, Zhang Y, Chen Y. Icotinib-resistant HCC827 cells produce exosomes with mRNA MET oncogenes and mediate the migration and invasion of NSCLC. Respir Res. 2019;20:217. 10.1186/s12931-019-1202-z PMC6790059 31606039

[176] Hoshino A, Costa-Silva B, Shen T-L, Rodrigues G, Hashimoto A, Mark MT, Molina H, Kohsaka S, Di Giannatale A, Ceder SJN: Tumour exosome integrins determine organotropic metastasis. Nature. 2015;527:329–35. 10.1038/nature15756 PMC4788391 26524530

[177] Fong MY, Zhou W, Liu L, Alontaga AY, Chandra M, Ashby J, Chow A, O’Connor STF, Li S, Chin ARJNcb: Breast-cancer-secreted miR-122 reprograms glucose metabolism in premetastatic niche to promote metastasis. Nat Cell Biol. 2015;17:183–94. 10.1038/ncb3094 PMC4380143 25621950

[178] Becker A, Thakur BK, Weiss JM, Kim HS, Peinado H, Lyden D (2016). Extracellular vesicles in cancer: cell-to-cell mediators of metastasis. Cancer Cell.

[179] Baietti MF, Zhang Z, Mortier E, Melchior A, Degeest G, Geeraerts A, Ivarsson Y, Depoortere F, Coomans C, Vermeiren E (2012). Syndecan–syntenin–ALIX regulates the biogenesis of exosomes. Nat Cell Biol.

[180] Tai YL, Chen KC, Hsieh JT, Shen TL (2018). Exosomes in cancer development and clinical applications. Cancer Sci.

[181] Bastos N, Ruivo CF, da Silva S, Melo SA. Exosomes in cancer: Use them or target them? Semin Cell Dev Biol. 2018;78:13–21. 10.1016/j.semcdb.2017.08.009 28803894

[182] Ramani VC, Purushothaman A, Stewart MD, Thompson CA, Vlodavsky I, Au JLS, Sanderson RD (2013). The heparanase/syndecan-1 axis in cancer: mechanisms and therapies. FEBS J.

[183] Wu M, Wang G, Hu W, Yao Y, Yu X-F (2019). Emerging roles and therapeutic value of exosomes in cancer metastasis. Mol Cancer.

[184] Thompson CA, Purushothaman A, Ramani VC, Vlodavsky I, Sanderson RD (2013). Heparanase regulates secretion, composition, and function of tumor cell-derived exosomes. J Biol Chem.

[185] Sento S, Sasabe E, Yamamoto T (2016). Application of a persistent heparin treatment inhibits the malignant potential of oral squamous carcinoma cells induced by tumor cell-derived exosomes. PLoS One.

[186] Nishida-Aoki N, Tominaga N, Takeshita F, Sonoda H, Yoshioka Y, Ochiya T (2017). Disruption of circulating extracellular vesicles as a novel therapeutic strategy against cancer metastasis. Mol Ther.

[187] de la Fuente A, Alonso-Alconada L, Costa C, Cueva J, Garcia-Caballero T, Lopez-Lopez R, Abal M. M-trap: exosome-based capture of tumor cells as a new technology in peritoneal metastasis. J Natl Cancer Institute. 2015;107:djv184. 10.1093/jnci/djv184 PMC4836824 26150590

[188] Xie X, Nie H, Zhou Y, Lian S, Mei H, Lu Y, Dong H, Li F, Li T, Li B (2019). Eliminating blood oncogenic exosomes into the small intestine with aptamer-functionalized nanoparticles. Nat Commun.

[189] Khodashenas Limoni S, Salimi F, Forouzandeh Moghaddam M (2017). Designing pLEX-LAMP-DARPin lentiviral vector for exression of HER2 targeted DARPin on exosome surface. J Mazandaran Univ Med Sci.

[190] Limoni SK, Moghadam MF, Moazzeni SM, Gomari H, Salimi F (2019). Engineered exosomes for targeted transfer of siRNA to HER2 positive breast cancer cells. Appl Biochem Biotechnol.

[191] Gomari H, Moghadam MF, Soleimani M (2018). Targeted cancer therapy using engineered exosome as a natural drug delivery vehicle. OncoTargets Therapy.

[192] Bhagwat N, Dulmage K, Pletcher CH, Wang L, DeMuth W, Sen M, Balli D, Yee SS, Sa S, Tong F (2018). An integrated flow cytometry-based platform for isolation and molecular characterization of circulating tumor single cells and clusters. Sci Rep.

[193] Gorgannezhad L, Umer M, Islam MN, Nguyen N-T, Shiddiky MJJLoaC. Circulating tumor DNA and liquid biopsy: opportunities, challenges, and recent advances in detection technologies. Lab Chip. 2018;18:1174–96. 10.1039/C8LC00100F 29569666

[194] Snyder MW, Kircher M, Hill AJ, Daza RM, Shendure J (2016). Cell-free DNA comprises an in vivo nucleosome footprint that informs its tissues-of-origin. Cell.

[195] Fleischhacker M, Schmidt B (2007). Circulating nucleic acids (CNAs) and cancer—a survey. Biochimica et Biophysica Acta (BBA)-Reviews on Cancer.

[196] Thierry A, El Messaoudi S, Gahan P, Anker P, Stroun M (2016). Origins, structures, and functions of circulating DNA in oncology. Cancer Metastasis Rev.

[197] Bronkhorst AJ, Ungerer V, Holdenrieder S (2019). The emerging role of cell-free DNA as a molecular marker for cancer management. Biomol Detect Quantif.

[198] Fűri I, Kalmár A, Wichmann B, Spisák S, Schöller A, Barták B, Tulassay Z, Molnár B (2015). Cell free DNA of tumor origin induces a 'Metastatic' expression profile in HT-29 Cancer cell line. PLoS One.

[199] Lee K-H, Shin T-J, Kim W-H, Cho J-Y (2019). Methylation of LINE-1 in cell-free DNA serves as a liquid biopsy biomarker for human breast cancers and dog mammary tumors. Sci Rep.

[200] Thakur BK, Zhang H, Becker A, Matei I, Huang Y, Costa-Silva B, Zheng Y, Hoshino A, Brazier H, Xiang J (2014). Double-stranded DNA in exosomes: a novel biomarker in cancer detection. Cell Res.

[201] Yokoi A, Villar-Prados A, Oliphint PA, Zhang J, Song X, De Hoff P, Morey R, Liu J, Roszik J, Clise-Dwyer K, et al. Mechanisms of nuclear content loading to exosomes. Sci Adv. 2019;5:eaax8849. 10.1126/sciadv.aax8849 PMC6867874 31799396

[202] Kostyuk SV, Ermakov AV, Alekseeva AY, Smirnova TD, Glebova KV, Efremova LV, Baranova A, Veiko NN (2012). Role of extracellular DNA oxidative modification in radiation induced bystander effects in human endotheliocytes. Mutat Res.

[203] Dvořáková M, Karafiát V, Pajer P, Kluzáková E, Jarkovská K, Pekova S, Krutílkova L, Dvořák M. DNA released by leukemic cells contributes to the disruption of the bone marrow microenvironment. Oncogene. 2013;32:5201–9. 10.1038/onc.2012.553 23222712

[204] Garcia-Olmo DC, Picazo MG, Garcia-Olmo D (2012). Transformation of non-tumor host cells during tumor progression: theories and evidence. Expert Opin Biol Ther.

[205] Garcia-Olmo DC, Dominguez C, Garcia-Arranz M, Anker P, Stroun M, Garcia-Verdugo JM, Garcia-Olmo D (2010). Cell-free nucleic acids circulating in the plasma of colorectal cancer patients induce the oncogenic transformation of susceptible cultured cells. Cancer Res.

[206] Mittra I, Samant U, Sharma S, Raghuram GV, Saha T, Tidke P, Pancholi N, Gupta D, Prasannan P, Gaikwad A, et al. Cell-free chromatin from dying cancer cells integrate into genomes of bystander healthy cells to induce DNA damage and inflammation. Cell Death Disc. 2017;3:17015. 10.1038/cddiscovery.2017.15 PMC5447133 28580170

[207] Wan JC, Massie C, Garcia-Corbacho J, Mouliere F, Brenton JD, Caldas C, Pacey S, Baird R, Rosenfeld N (2017). Liquid biopsies come of age: towards implementation of circulating tumour DNA. Nat Rev Cancer.

[208] Mouliere F, El Messaoudi S, Pang D, Dritschilo A, Thierry AR (2014). Multi-marker analysis of circulating cell-free DNA toward personalized medicine for colorectal cancer. Mol Oncol.

[209] Liebs S, Keilholz U, Kehler I, Schweiger C, Haybäck J, Nonnenmacher A (2019). Detection of mutations in circulating cell-free DNA in relation to disease stage in colorectal cancer. Cancer Med.

[210] Sanchez C, Snyder MW, Tanos R, Shendure J, Thierry AR (2018). New insights into structural features and optimal detection of circulating tumor DNA determined by single-strand DNA analysis. NPJ Genomic Med.

[211] Chang Y, Tolani B, Nie X, Zhi X, Hu M, He B (2017). Review of the clinical applications and technological advances of circulating tumor DNA in cancer monitoring. Ther Clin Risk Manag.

[212] Oellerich M, Schütz E, Beck J, Kanzow P, Plowman PN, Weiss GJ, Walson PD (2017). Using circulating cell-free DNA to monitor personalized cancer therapy. Crit Rev Clin Lab Sci.

[213] Barbosa A, Peixoto A, Pinto P, Pinheiro M, Teixeira MR. Potential clinical applications of circulating cell-free DNA in ovarian cancer patients. Expert Rev Mol Med. 2018;20:E6. 10.1017/erm.2018.5 30558693

[214] Kustanovich A, Schwartz R, Peretz T, Grinshpun A. Life and death of circulating cell-free DNA. Cancer Biol Therapy. 2019;20:1057–1067. 10.1080/15384047.2019.1598759 PMC6606043 30990132

[215] Wang R, Li X, Zhang H, Wang K, He J (2017). Cell-free circulating tumor DNA analysis for breast cancer and its clinical utilization as a biomarker. Oncotarget.

[216] Cristiano S, Leal A, Phallen J, Fiksel J, Adleff V, Bruhm DC, Jensen SO, Medina JE, Hruban C, White JR (2019). Genome-wide cell-free DNA fragmentation in patients with cancer. Nature.

[217] Caruso S, Poon IK (2018). Apoptotic cell-derived extracellular vesicles: more than just debris. Front Immunol.

[218] Wickman G, Julian L, Olson M (2012). How apoptotic cells aid in the removal of their own cold dead bodies. Cell Death Differ.

[219] Ogden CA, de Cathelineau A, Hoffmann PR, Bratton D, Ghebrehiwet B, Fadok VA, Henson PM (2001). C1q and mannose binding lectin engagement of cell surface calreticulin and CD91 initiates macropinocytosis and uptake of apoptotic cells. J Exp Med.

[220] Julian L, Olson MF (2015). Apoptotic membrane dynamics in health and disease. Cell Health Cytoskeleton.

[221] Xu X, Lai Y, Hua Z-C. Apoptosis and apoptotic body: disease message and therapeutic target potentials. Biosci Rep. 2019;39:BSR20180992. 10.1042/BSR20180992 PMC6340950 30530866

[222] Gordon S, Plüddemann A (2018). Macrophage clearance of apoptotic cells: a critical assessment. Front Immunol.

[223] Bergsmedh A, Szeles A, Henriksson M, Bratt A, Folkman MJ, Spetz A-L, Holmgren L (2001). Horizontal transfer of oncogenes by uptake of apoptotic bodies. Proc Natl Acad Sci.

[224] Samos J, García-Olmo DC, Picazo MG, Rubio-Vitaller A, García-Olmo D (2006). Circulating nucleic acids in plasma/serum and tumor progression. Ann N Y Acad Sci.

[225] Hulea L, Gravel S-P, Morita M, Cargnello M, Uchenunu O, Im YK, Lehuédé C, Ma EH, Leibovitch M, McLaughlan S (2018). Translational and HIF-1α-Dependent Metabolic Reprogramming Underpin Metabolic Plasticity and Responses to Kinase Inhibitors and Biguanides. Cell Metabolism.

[226] Campbell SL, Wellen KE (2018). Metabolic signaling to the nucleus in cancer. Mol Cell.

[227] Buck MD, Sowell RT, Kaech SM, Pearce EL (2017). Metabolic instruction of immunity. Cell.

[228] Sanford-Crane H, Abrego J, Sherman MH (2019). Fibroblasts as modulators of local and systemic Cancer metabolism. Cancers.

